# The interaction of MYC with the trithorax protein ASH2L promotes gene transcription by regulating H3K27 modification

**DOI:** 10.1093/nar/gku312

**Published:** 2014-04-29

**Authors:** Andrea Ullius, Juliane Lüscher-Firzlaff, Ivan G. Costa, Gesa Walsemann, Alexandra H. Forst, Eduardo G. Gusmao, Karsten Kapelle, Henning Kleine, Elisabeth Kremmer, Jörg Vervoorts, Bernhard Lüscher

**Affiliations:** 1 Institute of Biochemistry and Molecular Biology, Medical School, RWTH Aachen University, 52074 Aachen, Germany; 2 IZKF Research Group Computational Biology, Medical School, RWTH Aachen University, 52074 Aachen, Germany; 3 Helmholtz Zentrum München, Institut für Molekulare Immunologie, Marchioninistr. 25, 81377 München, Germany

## Abstract

The appropriate expression of the roughly 30,000 human genes requires multiple layers of control. The oncoprotein MYC, a transcriptional regulator, contributes to many of the identified control mechanisms, including the regulation of chromatin, RNA polymerases, and RNA processing. Moreover, MYC recruits core histone-modifying enzymes to DNA. We identified an additional transcriptional cofactor complex that interacts with MYC and that is important for gene transcription. We found that the trithorax protein ASH2L and MYC interact directly *in vitro* and co-localize in cells and on chromatin. ASH2L is a core subunit of KMT2 methyltransferase complexes that target histone H3 lysine 4 (H3K4), a mark associated with open chromatin. Indeed, MYC associates with H3K4 methyltransferase activity, dependent on the presence of ASH2L. MYC does not regulate this methyltransferase activity but stimulates demethylation and subsequently acetylation of H3K27. KMT2 complexes have been reported to associate with histone H3K27-specific demethylases, while CBP/p300, which interact with MYC, acetylate H3K27. Finally WDR5, another core subunit of KMT2 complexes, also binds directly to MYC and in genome-wide analyses MYC and WDR5 are associated with transcribed promoters. Thus, our findings suggest that MYC and ASH2L–KMT2 complexes cooperate in gene transcription by controlling H3K27 modifications and thereby regulate bivalent chromatin.

## INTRODUCTION

The oncoprotein MYC functions as a transcriptional regulator. Together with its heterodimerization partner MAX, MYC controls the expression of many genes with broad physiological implications (1–4). MYC impacts gene expression at multiple levels, including the recruitment of several transcriptional cofactors that have the potential to affect the state of chromatin and the activities of polymerase complexes. Among these cofactors are different acetyltransferases that modify core histones as well as other proteins associated with the transcriptional machinery and thus promote gene expression (5–8). Moreover, MYC recruits P-TEFb, a complex that includes cyclin-dependent kinase 9 and cyclin T1, which phosphorylates serine 2 of the C-terminal domain (CTD) of RNA polymerase II (POL II), stimulating its activity (9–11). CDK7, a TFIIH subunit and possibly CDK8 as subunit of a mediator complex are additional kinases that are recruited by MYC to control POL II activity (12,13). Together these findings suggest that MYC is capable of influencing chromatin structure and polymerase activity by multiple means, resulting in regulation of gene transcription (3).

The modification of chromatin, i.e. of DNA and DNA-associated proteins, by various chemical groups controls the accessibility of factors to DNA that in turn influence DNA-related processes, such as transcription, repair and replication. Key targets during these processes are nucleosomes (14–16). The core histones, which form the proteinaceous center of the nucleosome, are the target of multiple enzymes. Post-translational modifications of core histones and the exchange with histone variants modulate nucleosomal stability and the interaction with numerous proteins. More than 100 different modifications of core histones are presently known. While for some of these modifications functional consequences are described, many have not been studied in great detail. Also the combinatorial effects of these modifications are only partially understood. Methylation of lysine 4 of histone H3 (H3K4) is associated with accessible chromatin (17). This residue can be mono-, di- or trimethylated by different enzymes, including the mixed-lineage leukemia (MLL) and the SET1A/B methyltransferases (MTase). These MTases have been classified as the KMT2 (lysine-specific methyltransferases) group of lysine-specific MTases (18). Both the MLL and SET1A/B proteins are part of larger complexes (here referred to as KMT2 complexes), which are related to the yeast COMPASS complex (19). MLL1 and the closely related MLL2 are the orthologs of the *D. melanogaster* trithorax, a member of the evolutionary conserved trithorax group (trxG) family of proteins, which positively control gene transcription (20). In addition to the MTases, core subunits of these complexes include ASH2L, DPY30, RbBP5 and WDR5, which are required for catalytic activity of the MTase complexes (21–27). Ash2 was also identified in *D. melanogaster* as a trxG family member (28,29). Additional KMT2 complex subunits have been detected in various systems and under different experimental conditions, suggesting that the core subunits contribute to many different, more sophisticated complexes (17,19).

H3K4 monomethylation (H3K4me1) and H3K4 trimethylation (H3K4me3) serve as marks for enhancer regions and accessible promoters, respectively (30–32). H3K4me3 is associated with open chromatin at both active and poised promoters (33), indicating that additional determinants are required to specify the expression of H3K4me3 marked genes. Indeed, one critical histone mark that cooperates with H3K4me3 at promoters is the modification of H3K27 by acetylation (H3K27ac) (17).

We identified ASH2L in a screen for MYC-interacting proteins (34). We found that the two proteins interact directly in the nucleus and at specific promoters and that MYC recruits H3K4 MTase activity dependent on ASH2L. At the promoters studied MYC does not recruit ASH2L complexes; rather ASH2L is associated with MYC-regulated promoters prior to MYC binding. Moreover, MYC does not affect H3K4me3, despite the direct interaction with ASH2L. Instead binding of MYC alters acetylation of core histones through p300/CBP, as reported previously (8). MYC and ASH2L complexes together result in demethylation of H3K27 and subsequent acetylation, resulting in induced gene expression. Thus, MYC and ASH2L seem to cooperate in controlling distinct histone modifications that are associated with gene expression.

## MATERIALS AND METHODS

### Cell culture and transfections

All cell lines were incubated in humidified atmosphere at 37°C with 5% CO_2_. P493-6 and Jurkat T cells were cultivated in RPMI medium and HEK293, HEK293T, U2OS and HeLa cells were grown in DMEM+Glutamax medium, both supplemented with 10% heat-inactivated fetal calf serum and 1% penicillin/streptomycin (P/S). For growth-arrest and MYC suppression, P493-6 B cells were seeded at 5 × 10^5^/ml and treated with 0.1 μg/ml tetracycline for 72 h. To induce MYC expression, tetracycline was removed by washing the cells twice with phosphate buffered saline (PBS) and adding fresh RPMI medium containing 10% FCS (+MYC). Control cells were maintained in tetracycline-containing medium until harvest (-MYC).

Transfections of plasmids were carried out using the calcium phosphate method (35,36). Experiments were harvested 36 to 48 h after transfection when only protein-expressing plasmids were used. Experiments with plasmids expressing short-hairpin RNAs (pSuper) were harvested 72 h after transfection. Transient transfections with Dharmacon siRNAs were carried out using HiPerfect (Qiagen, Hilden Germany) transfection reagent according to the manufacturer's instructions at a final concentration of 15–20 nM.

Dharmacon siRNA pools (Thermo Scientific)

Control: siGENOME Non-Targeting siRNA Pool #2 (D-001206-14)

ASH2L: SMARTpool siGENOME ASH2L siRNA (M-019831-01)

MYC: SMARTpool siGENOME MYC siRNA (M-003282-07)

Dharmacon Set of 4 single siRNAs (Thermo Scientific)

ASH2L: siGENOME ASH2L siRNA (MQ-019831-01)

MYC: siGENOME MYC siRNA (MQ-003282-07)

The four individual siOligos were tested for knockdown efficiency (data not shown). The two most efficient siOligos were then used in additional control experiments (shown in Supplementary Figures S6 and S7).

### Plasmids, siRNAs and recombinant proteins

pcDNA3-Flag-MYC-1-439 (wt), -1-410, -1-350, -1-180, -101-439, -148-439, -178-439, -Δ103-263, -Δ265-329, -Δ265-367, -Δ319-341 and pCGN-HA-MYC-1-439 (wt), -1-366, -1-293, -1-220, -221-439, -294-439, -367-439 were kindly provided by W. Tansey (37). Plasmids expressing ASH2L were described previously (38). Mutants were generated using standard cloning techniques. The following pSuper (39) constructs were used: pSuper-ASH2L 5’-GGATCTCACTTACCGCCCT; pSuper-Control (ASH2Lmut) 5’-CCCTGCAGATCCATGCTT. pcDNA3-Flag-MLL2 653 (Addgene 11017), pcDNA3-Flag-WDR5 (Addgene 15552) and pcDNA3 RbBP5 (Addgene 15550) plasmids were used for the expression of the KMT2 complex components.

Recombinant proteins were produced in *Escherichia coli* (*E. coli*) BL21(DE)pLysS as GST-fusion or His_6_-fusion proteins using the following constructs pGEX4T3-ASH2L-wt, -1-121, -1-279, -1-394, -1-444, pGEX2T-MYC-1-262 (N262), -1-156, -263-439, pGEX3X-MAX, pGEX2T-YY1, pGEX4T3-H3-N, pQE30-ASH2-1-387, pRSET-His_6_-WDR5 (40). Transformed bacteria were grown to an OD of 0.7–0.9 and protein expression was induced with 0.4 mM IPTG either at 37°C for 4 h or at 21°C for 16 h. Bacterial pellets were lysed in buffer A (50 mM Tris-HCl, pH 8.0, 100 mM NaCl, 0.5% (v/v) Nonidet P40, 5 mM EDTA, 10 mM DTT, 0.2 mM phenylmethylsulphonyl fluoride (PMSF), 1% (v/v) Aprotinin), sonicated, and centrifuged at 10.000 x g at 4°C for 30 min. GST-fusion proteins were separated from the cell lysate with glutathione-agarose (Sigma-Aldrich) and eluted from the beads with 10 mM glutathione in buffer A.

Maltose-binding protein-MYC fusion (MBP-MYC) and MBP (41) were purified from *E. coli* BL21(DE)pLysS grown in NZC-medium (1% NZ-Amine A, 0.5% (w/v) NaCl, 0.2% (w/v) MgCl_2_, 0.2% glucose) induced at OD 0.5 with 0.3 mM IPTG at 30°C for 4 h. Cells were lysed in buffer C (20 mM Tris-HCl, pH 8.0, 200 mM NaCl, 1 mM EDTA, 1 mM DTT, 0.2 mM PMSF, 1% (v/v) Aprotinin), sonicated, and centrifuged at 10,000 x g at 4°C for 30 min. MBP proteins were separated from the cell lysate with amylose-agarose (New England Biolabs) and eluted from the beads with 10 mM maltose in buffer A. Core histones and recombinant histone H3 (New England Biolabs) and GST-Histone H3 N’-terminal-tails (provided by Y. Shinkai) (40) were used as substrates.

### GST-pull-down assays

For binding reactions, 3 μg GST or GST-fusion proteins were bound to glutathione–agarose beads and incubated with [^35^S]methionine-labeled, *in vitro* transcribed and translated proteins in binding buffer or with 3 μg of recombinant His_6_-fusion proteins (42). For *in vitro* translation/transcription, the TNT Quick Coupled Transcription/Translation System (Promega) was used with the plasmids pcDNA3-Flag-MYC and pcDNA3-Flag-ASH2L.

### Co-immunoprecipitation

For in cell interaction assays, whole-cell lysates from 2 × 10^7^ cells were prepared in F-buffer (10 mM Tris-HCl, pH 7.05, 50 mM NaCl, 30 mM Na_3_PO_4_, 50 mM NaF, 5 μM ZnCl, 100 μM Na_3_VO_4_, 1% Triton X-100, 1 mM PMSF, 5 units/ml α2-macroglobulin, 2.5 units/ml pepstatin A, 2.5 units/ml leupeptin, 0.15 mM benzamidin). Co-immunoprecipitated proteins were detected by western blot analysis (43,44).

### Histone methyltransferase assay

Whole-cell lysates from 3 × 10^7^ cells were prepared in F-buffer to immunoprecipitate MYC or ASH2L and the associated MTase. MYC- and ASH2L-associated MTase was measured with 5 μg core histones or GST-H3 N-terminal tails as substrates in the presence of 1.5 μl [^3^H]-SAM (Perkin-Elmer Life Sciences, NET-155H, 0.55 mCi/ml, 79.8 Ci/mmol) at 30°C for 30 min. Modified proteins were visualized by SDS-PAGE and autoradiogaphy. Site specificity and MYC-associated methyltransferase activity was measured with 1 μg recombinant histone H3 in the presence of 100 μM SAM at 30°C for 1 h. Histone H3 methylation was detected by immunoblotting with different H3K4 and H3K9 methyl-specific antibodies.

### Immunofluorescence staining

HeLa cells were seeded onto coverslips, transiently transfected with pSuper-ASH2L and fixed in PBS containing 3.7% paraformaldehyde 72 h after transfection. Cells were permeabilized in PBS with 0.2% Triton X-100 at room temperature for 5 min and stained with the ASH2L-specific rat mAb 4C5 and the H3K4me3-specific rabbit polyclonal antibody ab8898 (abcam) at 37°C for 45 min. Anti-rat-Cy3 and anti-rabbit-Cy2 secondary antibodies were used at 37°C for 30 min. Coverslips were mounted with Moviol (Merck) in PBS containing 2.5% N-propylgallate (Sigma). For the proximity ligation assay (PLA), the DuolinkII fluorescence system was used in conjunction with the Probemaker Plus kit for labeling of the secondary anti-rat antibody according to the manufacturer's instructions (Olink Bioscience) (45).

### Antibodies

The following antibodies were used for IP, chromatin immunoprecipitations (ChIP), and western blot: rabbit anti-MYC (N262, sc-764; C19, sc-788, both Santa Cruz; IG13, 022Y, 1236 all three provided by L.-G. Larsson), rabbit anti-MAX (C17, sc-197, Santa Cruz), rat anti-MXD1 (5F4), mouse anti-nucleolin (MS-3, sc-8031, Santa Cruz) rabbit anti-ASH2L (A300–489A, Bethyl; sera 548 and 549 (antigen ASH2L-1-444)), rat anti-ASH2L (4C5 and 4B5 (antigen ASH2L-1-279)), mouse anti-WDR5 (ab56919, abcam), rabbit anti-RbBP5 (A300-109A, Bethyl), rabbit anti-H3 (ab1791, abcam), rabbit anti-H3ac (06-599, Millipore), rabbit anti-H3K9ac (ab4441, abcam), rabbit anti-H3K27ac (ab4729, abcam), rabbit anti-H3K4me3 (ab8580, abcam), rabbit anti-H3K4me2 (07-030, Millipore), rabbit anti-H3K4me1 (ab8895, abcam), rabbit anti-H3K9me3 (ab8898, abcam), rabbit H3K9me2 (07-212, Millipore), rabbit anti-H3K9me1 (ab9045, abcam), rabbit anti-H3K27me3 (ab6002, abcam), rabbit anti-CBP (C-20, sc-583, Santa Cruz), rabbit anti-p300 (N-15, sc-584, Santa Cruz), rabbit IgG (Kch-504-250, Diagenode), mouse anti-actin (MP Biomedicals), rat anti-HA (3F10, Roche), mouse anti-Flag (M2, Sigma-Aldrich), rabbit anti-caspase 5 (2157, was kindly provided by A. Krippner-Heidenreich).

### ChIP- and Re-ChIP-qPCR

ChIP-qPCR assays were performed with the Diagenode OneDay ChIP kit according to the manufacturer's instructions. P493-6 and HEK293T cells were cross-linked with 1% formaldehyde (by adding 37% formaldehyde to the medium) on a shaking platform for 10 min at room temperature and quenched for 5 min by adding 125 mM glycine. Cells were lysed in 100 μl lysis buffer (2 × 10^7^ P493-6 cells and 1×10^7^ HEK293T cells) and chromatin was sheared for 15 min using a Bioruptor (Diagenode). Immunoprecipitation was performed with 100 μg chromatin (input) and 2 μg antibody. DNA was purified and the quantitative polymerase chain reaction (qPCR) was performed with the following primer pairs:

*Ctrl* pp4 (for): 5’-GCGTGACTCAAATTGTGTGTGCCT-3’
*Ctrl* pp4 (rev): 5’-ATCAAGCATTCAGCAGCGTTC CA-3’
*CCND2 prom1* (for): 5’-CCCCTTCCTCCTGGAGTGAAATAC-3’
*CCND2 prom2* (rev): 5’-CGTGCTCTAACGCATCCTTGAGTC-3’
*CCND2 Ex1* (for): 5’-GGGAGAGCGAGACCAGTTTTAAG-3’
*CCND2 Ex1* (rev): 5’-CCTTTGGCTAAATAGGGGGTTTTC-3’
*ODC E-box* (for): 5’-ATCACTTCCAGGTCCCTTGCAC-3’
*ODC E-box* (rev): 5’-TTCCACCTGGCGTTCAGTACCT-3’
*NCL In1* (for): 5’-TGGGCCGGGAAATGGCGGTA-3’
*NCL In1* (rev): 5’-TAGGCCACCACGTGCCCGAA-3’

For Re-ChIP experiments the first IP was performed with 100–200 μg chromatin (input). Chromatin was released from beads using release buffer (TE buffer, pH 7.5, 1% SDS, 10 mM DTT) for 30 min at 37°C and diluted in 4 volumes ChIP buffer (Diagenode OneDay ChIP kit, with protease inhibitor cocktail and 10 mM iodoacetamide). Then, 2 μg of antibody were added for the second immunoprecipitation, followed by DNA purification and qPCR. PCR was performed as described below.

The DNA was purified from one tenth of the input chromatin that was used for the ChIPs, diluted two-fold and used for qPCR amplification. The dilution factor was included in the % input calculation of all the qPCR results.

### qRT-PCR

Total RNA was extracted using RNeasy Mini Kit (Qiagen) and DNase digestion (Qiagen) according to the manufacturer's instructions. 1 μg of RNA was reverse transcribed into cDNA using the QuantiTect Reverse Transcription Kit (Qiagen) and analyzed by qPCR with SensiMix SYBR Green Mix (Bioline) in a Rotor-Gene Q (Qiagen) machine.

The following primer pairs were used for amplification:

*β-glucuronidase* (*GUS*) for: 5’-CTCATTTGGAATTTTGCCGATT-3’,
*β-glucuronidase* (*GUS*) rev: 5’-CCGAGTGAAGATCCCCTTTTTA-3’

QuantiTect primer assays (Qiagen): *MYC* (Hs_Myc_1_SG), *ASH2L* (Hs_ASH2L_1_SG), *CCND2* (Hs_CCND2_1_SG), *ODC* (Hs_ODC1_1_SG), *NCL* (Hs_NCL_1_SG). Relative mRNA expression was calculated by the comparative ΔΔCt method and normalized to the housekeeping gene *GUS* using Rotor-Gene Q software.

### FACS

P493-6 cells were harvested by centrifugation, washed with PBS and fixed by adding 100% methanol at -20°C. After RNase A treatment (20 μg/ml) cells were stained with propidium iodide (PI, 50 μg/ml) for 15 min at room temperature and analyzed using a FACSCanto II (BD Biosciences).

### Bioinformatics analysis of publicly available ChIP-Seq data

Public data from ChIP experiments followed by massively parallel DNA sequencing (ChIP-Seq) was used to analyze the occupancy of the factors MYC, WDR5 and POL II and the occurrence of the histone modifications H3K4me3, H3K4me1 and H3K27me3 in normal human epidermal keratinocytes (NHEK). Data from histone modifications, POL II, MYC and controls were obtained from the ENCODE project (GEO accession GSE29611, GSM748557). WDR5 ChIP-Seq was obtained from published findings (46) (GEO accession GSE42180). No read alignments were provided in GEO for WDR5 and MYC. For those, the alignments from original sequence reads were performed using Burrows-Wheeler Alignment tool with default parameters (47). All alignments were based on the human reference genome Hg19. Peak calling was performed with MACS (48) with a *p*-value of 10^−5^ and the ChIP-Seq control as input.

### Definition of regulatory features

Publicly available data for histone modifications were combined to define regulatory regions in NHEK cells: active promoters (both H3K27ac and H3K4me3 peaks overlapping with the core promoter, 200 bps upstream of a gene transcription start site (TSS)), active enhancers (regions distant from core promoters with peaks of both H3K4me1 and H3K27ac), poised promoters (H3K4me3 and H3K27me3 (49)), poised enhancers (H3K4me1, lack of H3K27ac (50)) and closed chromatin regions (only H3K27me3). Regions not fitting any of these criteria were indicated as ‘other’. bedTools (51) was used to find the regions of overlap between these sites that contain histone with specific modifications and the TSS of genes. Gene TSS positions were based on Hg19 RefSeq annotation obtained from USCD Table Browser. Additionally, the overlap of these regions with POL II ChIP-Seq peaks was evaluated. As a last step, the peaks from MYC, WDR5 and regions containing both MYC and WDR5 peaks were related to the above-mentioned regulatory features (see Supplementary Tables S1 and S2 for statistics).

### Analysis of transcription factor binding sites

To analyze the presence of binding sites for MYC and ASH2L inside ChIP-Seq peaks, the summit regions of the Chip-Seq peaks (positions with highest reads within a peak as provided by MACS), extended by 125 bps in each direction, were inspected. This procedure ensures that all peaks possess the same size and avoids biasing the binding site statistics. The choice of peaks with a size of 250 bps is in accordance with previously published work (52). Note that other choices of peak sizes (500, 100 and 80 bps) resulted in similar qualitative results (data not shown). As background, random genomic regions with the same characteristics of the ChIP-Seq peaks were obtained. The MYC position weight matrix (MA0147.1) from the Jaspar database (53) and the ASH2L position weight matrix from (54) were used. Biopython (55) was employed to perform binding site searches. Functions provided by the Motif class were applied to calculate a bit-score based on the application of a position Weight Matrix in the ChIP-Seq peaks. An empirical statistical test based on dynamic programming was used to define a bit-score threshold with a false discovery rate of 0.0001 (56). The number of MYC, WDR5 and both MYC and WDR5 ChIP-Seq peaks with at least one binding site motif were counted and a Fisher's Exact test performed to evaluate whether the proportion of peaks with binding sites is higher than in the background regions (Supplementary Table S3). Using the same analysis, but classifying the MYC or WDR5 peaks that belong to the previously described regulatory features, no relation between the proportion of binding sites and the regulatory features surrounding the peaks was detected (not shown). The analysis was performed with the Regulatory Genomics Toolbox available at https://code.google.com/p/reg-gen/.

### Analysis of gene expression

To evaluate if regions of ChIP-Seq peaks and NHEK expression data from RNA-Seq experiments are related, the alignment of RNA-Seq reads from the ENCODE project (GSM958736) was analyzed. The summits of peaks of MYC, WDR5 and both MYC and WDR5 were extended by 1000 bps to capture the expression of neighboring genes. The number of aligned reads inside the ChIP-Seq peaks was counted and normalized by the size of the peaks. In addition, the same analysis was performed considering only ChIP-Seq peaks overlapping with the region 200 bps upstream of a TSS.

## RESULTS

### MYC and ASH2L interact in cells

**Figure 1.**
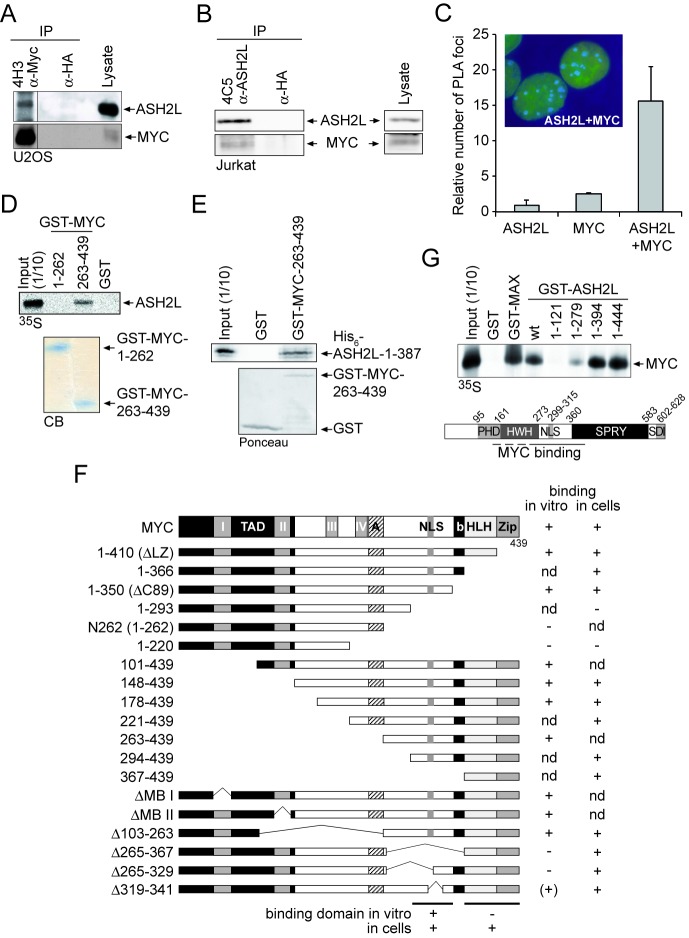
ASH2L interacts with MYC *in vitro* and in cells. (**A**) MYC was immunoprecipitated from U2OS cells using the mAb 4H3. HA-tag-specific antibodies served as control. The co-immunoprecipitation of ASH2L was analyzed by immunoblotting using the ASH2L-specific 4C5 mAb. MYC was detected using pAb N262. The different lanes were run on the same western blot. (**B**) ASH2L was immunoprecipitated from lysates of Jurkat T cells using the mAb 4C5. ASH2L was detected on western blots with pAb 548 and the co-immunoprecipitated MYC with MYC-specific N262 polyclonal antibodies. Antibodies specific for the HA-tag were used as negative control. (**C**) *In situ* PLA in U2OS cells using primary mAb 4C5 to detect ASH2L and primary N262 purified pAb to detect MYC and species-specific secondary antibodies with oligos attached to them (PLA probes). For negative control, one primary antibody was assayed with the species-specific secondary antibody. The number of foci were counted from 50 cells of three independent experiments, displayed as mean value and standard deviation (using students’ *t*-test). The inset shows two representative cells with foci in blue and the DNA stained in green. (**D**) GST-pull-down assays were carried out with different fragments of MYC (as indicated) fused to GST and GST alone as control. The binding of *in vitro* transcribed and translated, ^35^S-methionine-labeled ASH2L was analyzed by SDS-PAGE and autoradiography. The fusion proteins used are shown below in a Coomassie-Blue-stained gel (CB). (**E**) GST-pull-down assay of bacterially expressed and purified MYC and ASH2L fusion proteins were performed with GST-MYC-C176 containing the ASH2L interaction domain and with His_6_-ASH2L-N387 containing the N-terminal 387 amino acids that are sufficient for the interaction with MYC. (**F**) Summary of the interactions of ASH2L with MYC of *in vitro* pull-down assays and of co-immunoprecipitation experiments obtained from HEK293 cells upon transient expression of the respective fragments. (**G**) GST*-*pull-down assays were carried out with different fragments of ASH2L (as indicated) fused to GST and GST alone as control. The binding of *in vitro* transcribed and translated, ^35^S-methionine-labeled MYC was analyzed by SDS-PAGE and autoradiography. The bottom panel shows the schematic organization of ASH2L. PHD, atypical plant homeodomain; HWH, helix-winged-helix domain; NLS, nuclear localization signal; SPRY, an SP1a and RYanodine receptor domain; SDI, SDC1/DPY30 interaction motif (54,114–116). The results of the GST-pull-down assays are summarized schematically at the bottom.

In a previous study, we identified three proteins that interact with MYC (34). One of these proteins is ARTD10/PARP10, a mono-ADP-ribosyltransferase (57). The other two proteins were identified as ASH2L and nucleolin. ASH2L was of interest to us because it has been described as a component of KMT2 group MTase complexes (14,58,59). Moreover, we found that ASH2L possesses transforming activity together with Ha-RAS in rat embryo fibroblasts and that the ASH2L protein, but not the mRNA, is overexpressed in the majority of human tumors (38,60). In the following, we have studied the interaction of MYC with ASH2L. To verify our initial purification result, MYC was immunoprecipitated (IPed) from lysates of U2OS cells using MYC-specific mAbs. We detected ASH2L in these but not in the control immunoprecipitates (IPs) (Figure 1A) with mAbs that we generated and that detect ASH2L specifically (Supplementary Figure S1A and B). These MYC complexes also contained nucleolin (data not shown) and an interaction between ASH2L and nucleolin was also seen (Supplementary Figure S1C). Moreover, ASH2L was co-IPed from Jurkat T cells and HEK293 cells using MYC- and MAX-specific antibodies, MAX being the heterodimeric partner of MYC (Supplementary Figure 1C and D). In a reciprocal experiment, ASH2L was IPed and MYC was detected in the complex (Figure 1B). Together these findings suggest that MYC interacts with ASH2L in cell extracts. To address whether MYC and ASH2L are also in close proximity in cells, we performed PLAs (45,61). MYC- and ASH2L-containing structures were identified exclusively in cell nuclei (Figure 1C), further supporting an interaction of ASH2L with MYC.

To evaluate the interaction between MYC and ASH2L, we carried out glutathione-S-transferase (GST) pull-down assays. *In vitro* transcribed and translated ASH2L interacted with the C-terminal region of MYC (GST-MYC(263–439)), but not with an N-terminal transactivation domain (GST-MYC(1–262)) or with GST alone (Figure 1D). This interaction is direct as bacterially expressed and purified MYC and ASH2L fusion proteins containing the respective interaction domains (see below) were sufficient for binding (Figure 1E). To map the interaction domain in MYC for ASH2L more precisely, different MYC deletion mutants were tested for binding to GST-ASH2L (Supplementary Figure S2A and B, summarized in Figure 1F). These studies defined a region N-terminal of the basic region/helix-loop-helix/leucine zipper (bHLHZip) domain in MYC, encompassing amino acids 263–350, as the major *in vitro* interaction domain (Figure 1F). Next, we evaluated whether this domain was important for MYC–ASH2L interaction in cells. Surprisingly, MYCΔ265-367 was still able to bind ASH2L (Supplementary Figure S2C), suggesting that in cells this region is not essential. Because the C-terminal region of MYC encompassing amino acids 367–439 was sufficient to interact with ASH2L in cells (Supplementary Figure S2D), it appeared that also the HLHZip region of MYC was able to bind to ASH2L (summarized in Figure 1F), most likely through an indirect interaction. In contrast, the N-terminal transactivation domain of MYC was unable to co-immunoprecipitate ASH2L (Supplementary Figure S2D).

The interaction of MYC and ASH2L was confirmed in GST-ASH2L pull-down experiments that implicated a central region of ASH2L in binding to MYC (Figure 1G). In support, a fragment-containing amino acids 1-17/160-395 of ASH2L was sufficient to co-immunoprecipitate MYC (Supplementary Figure S2E). Moreover, we tested for binding of the two additional core components WDR5 and RbBP5 of KMT2 complexes to MYC in pull-down assays. While RbBP5, which binds to both WDR5 and ASH2L, did not interact, WDR5, which connects KMT2 proteins and RbBP5 (23), was pulled down by the C-terminal region of MYC (Supplementary Figure S3A). The interaction of MYC with WDR5 is direct as documented by using bacterially expressed and purified fusion proteins (Supplementary Figure S3B). In support of the *in vitro* interaction data, in co-IP experiments using tagged proteins the C-terminal fragment of MLL2, WDR5 and ASH2L were detected efficiently, while RbBP5 binding was very weak (Supplementary Figure S3C). This indicates that MYC has the capacity to interact with at least two components of the KMT2 core complex. The main conclusion from these assays was that two distinct regions in MYC contribute to ASH2L binding, i.e. amino acids 263–350 through direct protein–protein interaction and the HLHZip domain by an indirect mechanism (Figure 1F), while a central region of ASH2L bound to MYC.

### MYC interacts with a methyltransferase complex dependent on ASH2L

**Figure 2.**
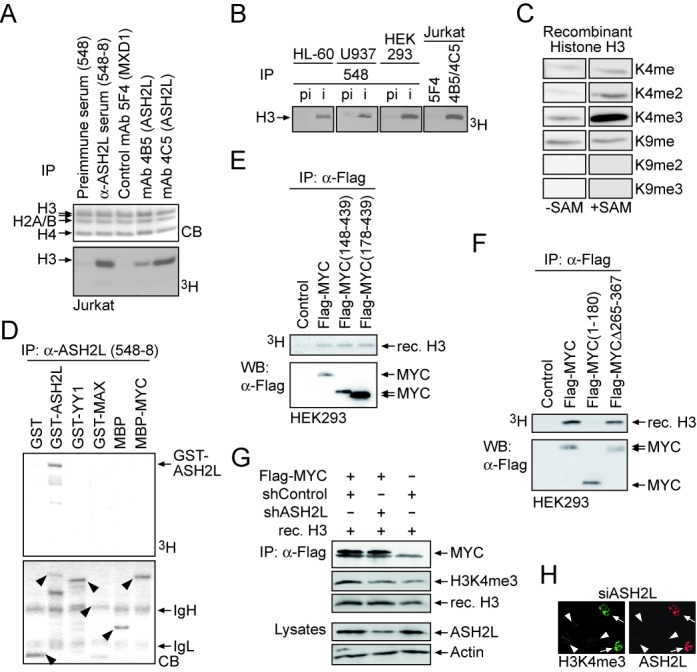
Recruitment of H3K4-specific methyltransferase activity by MYC is ASH2L dependent. (**A**) ASH2L was immunoprecipitated from Jurkat T cell F-buffer lysates with the indicated pre-immune and immune serum (548) and mAbs (4B5 and 4C5) and an isotype-specific control (5F4). The ASH2L-associated MTase activity was measured on core histones with the use of radiolabeled ^3^H-S-adenosylmethionine. The CB-stained gel shows the input of core histones. (**B**) ASH2L was immunoprecipitated from lysates of different cells using the ASH2L-specific 548 polyclonal antiserum (i) or the corresponding pre-immune serum (pi) or a combination of ASH2L-specific mAbs 4B5 and 4C5, and 5F4 for control. The ASH2L-associated MTase activity was measured on core histones with the use of radiolabeled ^3^H-S-adenosylmethionine. (**C**) The specificity of ASH2L-associated MTase activity from HEK293 was analyzed on recombinant histone H3 by using K4 and K9 methylation-specific antibodies as indicated. (**D**) ASH2L was immunoprecipitated from Jurkat T cell lysates. The indicated recombinant GST- or MBP-fusion proteins or GST and MBP alone as control were tested as substrates for ASH2L-associated MTase activity using ^3^H-S-adenosylmethionine. The arrowheads in the CB-stained gel indicate the input of the respective fusion protein. (**E**) and (**F**) Control vector, Flag-tagged MYC wt and mutants were expressed transiently in HEK293 cells, immunoprecipitated from F-buffer lysates using a Flag-specific antibody and the MYC-associated MTase activity was measured on recombinant GST-H3 N-terminal tails using ^3^H-S-adenosylmethionine. (**G**) HEK293T cells were transiently transfected with Flag-tagged MYC and pSuper constructs expressing an ASH2L-specific shRNA or a control shRNA. After immunoprecipitation of MYC from F-buffer lysates using a Flag-specific antibody, MYC-associated MTase activity was measured on recombinant Histone H3 using an H3K4me3-specific antibody. The expression of relevant proteins are shown for control. (**H**) HeLa cells were transiently transfected with pSuper constructs expressing either an ASH2L-specific shRNA or a control shRNA. The presence of ASH2L (mAb 4C5) and H3K4me3 (Abcam 8580) was visualized by immunofluorescence. Arrowheads and arrows indicate transfected and untransfected cells, respectively.

ASH2L is part of complexes that contain KMT2 MTases (18), which are specific for H3K4 (19). Therefore, we asked whether our ASH2L-specific antibodies were able to immunoprecipitate MTase activity. Indeed, the complexes isolated from lysates of different cell types with different monoclonal and polyclonal antibodies, but not control antibodies or pre-immune serum, possessed H3 MTase activity (Figure 2A and B). The specificity of these ASH2L complexes was analyzed with methylation-specific antibodies, demonstrating that the main activity was trimethylation of H3K4 (Figure 2C). These findings are consistent with previous analyses of human KMT2-containing complexes (25,62,63). Further evaluation of the substrate specificity of the complex revealed that neither MYC nor its partner MAX were modified by the ASH2L-associated MTase activity (Figure 2D). Similarly the transcriptional regulator YY1, which also interacts with MYC (64), was not modified. However, ASH2L itself was methylated in these *in vitro* experiments, pointing to a possible auto-regulatory mechanism.

The interaction of ASH2L with MYC suggested that MYC may recruit MTase activity. Indeed, MYC IPed from cells contained H3 MTase activity (Figure 2E). The recruitment of MTase activity was dependent on the presence of the CTD of MYC, whereas the N-terminal transactivation domain did not associate with an H3 MTase activity (Figure 2E and F). Deletion of the main *in vitro* ASH2L-binding domain in MYC was not sufficient to abrogate MTase activity. This is consistent with the observation that the HLHZip domain also bound ASH2L, albeit indirectly (Figure 2F), and that WDR5 also bound directly to MYC (Supplementary Figure 3). The MTase activity associated with MYC was specific for H3K4me3 and was largely dependent on the presence of ASH2L in cells (Figure 2G), consistent also with the finding that ASH2L is an essential component for the activity of KMT2 complexes (25,26). The knockdown of ASH2L reduced overall H3K4me3 levels (Figures 2H and 5B) (38). In summary, these findings suggest that MYC recruits a KMT2-type MTase activity through the interaction with ASH2L.

**Figure 3.**
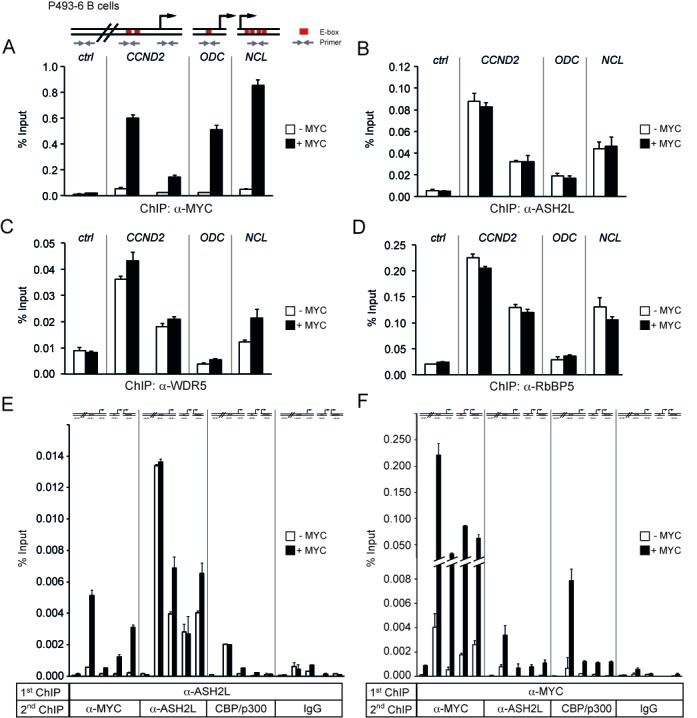
MYC and ASH2L interact at promoters. (**A**)–(**D**) P493-6 B cells were growth-arrested by the addition of tetracycline, which represses MYC expression, for 72 h. ChIP was performed six hours after either with (+MYC) or without removal of tetracycline (-MYC) using specific antibodies against MYC (A), ASH2L (B), WDR5 (C), or RbBP5 (D). Immunoprecipitated DNA was amplified by quantitative PCR (qPCR) with primers for *CCND2*, *ODC* and *NCL* promoter regions and a control region 22 kbp upstream of the *CCND2* promoter (*ctrl*), as indicated in the scheme. The E-boxes (red, known binding sites for MYC) in *ODC* and *NCL* are promoter proximal, the two E-boxes in *CCND2* are 1.2 kbp upstream of the transcriptional start site. (**E**) and (**F**) P493-6 B cells were treated as described for panel A. Sequential ChIP was carried out by first immunoprecipitating either ASH2L- (E) or MYC-containing (F) complexes, followed by a second ChIP with antibodies against MYC, ASH2L, CBP/p300, or IgG control. DNA fragments were amplified by qPCR as indicated for panel A. Error bars represent s.d. of PCR triplicates.

**Figure 4.**
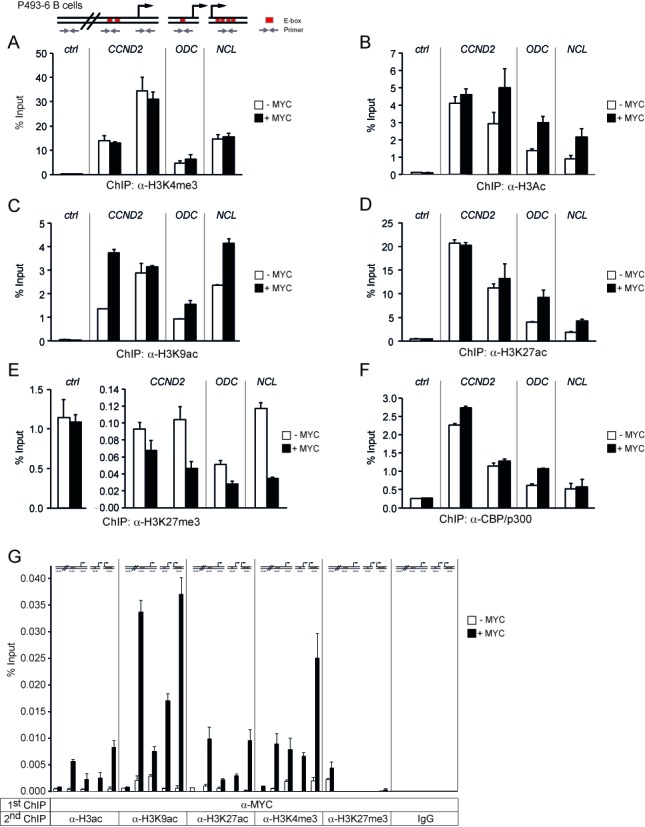
MYC stimulates histone H3 acetylation and inhibits H3K27 trimethylation but does not modulate H3K4 trimethylation. (**A**)–(**F**) P493-6 B cells were treated as described in the legend to Figure 3. ChIP was performed with antibodies against H3K4me3 (A), H3ac (B), H3K9ac (C), H3K27ac (D), H3K27me3 (E) and CBP/p300 (F). (**G**) Sequential ChIP was carried out by first immunoprecipitating MYC-containing complexes, followed by a second ChIP with antibodies against H3ac, H3K9ac, H3K27ac, H3K4me3, H3K27me3, or IgG control. The DNA fragments were amplified by qPCR with the primers indicated in the scheme. Error bars represent s.d. of PCR triplicates.

**Figure 5.**
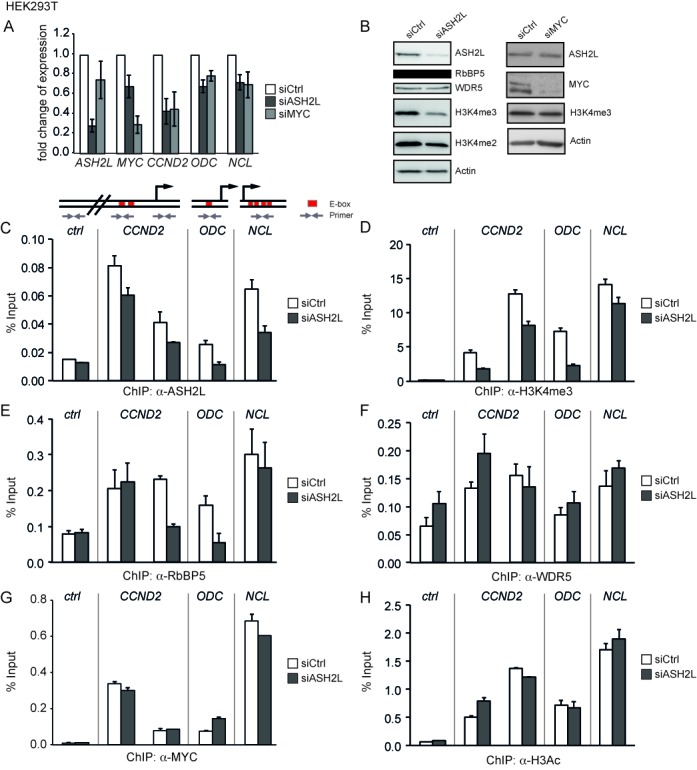
ASH2L is required for efficient gene expression and H3K4 trimethylation. (**A**) HEK293T cells were transiently transfected with siRNA oligo pools targeting *ASH2L* or *MYC* mRNA or a control oligo pool (*Ctrl*). Expression of *ASH2L*, *MYC*, *CCND2*, *ODC* and *NCL* mRNA was analyzed by quantitative RT-PCR and normalized to *GUS*. Error bars represent s.d. (*n* = 4). (**B**) Cell lysates of HEK293T cells from an experiment shown in panel A were used for western blot analyses with the indicated antibodies and actin as loading control. (**C**)–(**H**) HEK293T cells were transiently transfected with siRNA pools targeting *ASH2L* mRNA. Subsequent ChIP experiments were performed with antibodies against ASH2L (C), H3K4me3 (D), RbBP5 (E), WDR5 (F), MYC (G) and H3ac (H). DNA fragments were analyzed as described in the figures before. Error bars represent s.d. of PCR triplicates.

### MYC and ASH2L interact at promoters

One hypothesis derived from the findings described above is that MYC recruits ASH2L-containing complexes to E-boxes and MYC-controlled promoters. To address this hypothesis, we tested whether ASH2L complexes are recruited to promoters by MYC. In P493-6 B cells, a *MYC* transgene is under the control of a tetracycline regulatable promoter and controls proliferation of these cells (65,66). The addition of tetracycline provoked repression of *MYC* and accumulation of the cells in the G0/G1 phase of the cell cycle (Supplementary Figure S4A and B). Upon removal of tetracycline, *MYC* expression, both mRNA and protein, was reactivated and the cells entered the cell cycle (Supplementary Figure S4A and B). In parallel, the expression of three known MYC target genes, *CCND2* (encoding cyclin D2), *ornithine decarboxylase* (*ODC*) and *NCL* (encoding nucleolin), was induced (Supplementary Figure S4C) (6,67–71), demonstrating that these cells responded appropriately to MYC.

To evaluate the possible interaction of MYC and ASH2L on MYC-regulated promoters, we performed ChIP. MYC binding to the promoters of *CCND2*, *ODC* and *NCL* was induced upon removal of tetracycline, while no binding was detectable to a control region 22 kb upstream of the *CCND2* promoter (Figure 3A). However, ASH2L binding was not different between control resting and MYC-induced cells (Figure 3B), suggesting that MYC is not required for ASH2L to bind to these three MYC-regulated promoters. Not only ASH2L but also the ASH2L complex subunits RbBP5 and WDR5 were bound to all three promoters (Figure 3C and D). Their binding was largely independent of MYC with the exception of WDR5, which was slightly induced, possibly because it also directly interacts with MYC (Supplementary Figure S3). For control, in all experiments IgG ChIP were performed, which demonstrated that the obtained signals were specific (Supplementary Figure S5). The pattern of binding of the three KMT2 complex components was comparable to the different sites analyzed with binding to the *ODC* promoter being the lowest (Figure 3B–D).

Because MYC was not responsible for the recruitment of ASH2L-containing complexes, it was possible that MYC and ASH2L did not interact at the promoters analyzed, despite the fact that the two proteins were co-IPed from whole cell extracts, co-localize in nuclei and interact directly (Figure 1). Therefore, we performed re-ChIP experiments using antibodies specific for ASH2L and MYC as well as IgG as control. At all three promoters, but not at the genomic control site, ASH2L and MYC were present simultaneously upon induction of MYC (Figure 3E and F). Although these re-ChIP assays do not formally demonstrate that the two proteins directly interact, the results, together with the findings shown above, strongly suggest that MYC and ASH2L interact at MYC-regulated promoters.

### MYC does not affect H3K4 trimethylation

Although MYC did not affect the binding of the ASH2L complex to promoters, it was possible that MYC modulated the activity of the complex. Therefore, we analyzed H3K4me3, which was high at the promoters compared to the *CCND2* upstream control region but was not induced further by MYC (Figure 4A). Thus, MYC did neither regulate the recruitment of the ASH2L complex nor its associated MTase activity.

MYC has been shown to interact with different lysine-specific acetyltransferases (KATs) and to stimulate acetylation of core histones (3). Indeed, the induction of MYC in P493-6 B cells stimulated overall acetylation of histone H3 and more specifically acetylation of H3K9 and H3K27 at all three promoters, although not all the chromatin regions analyzed responded equally (Figure 4B–D). H3K4me3 and H3K27ac together define active promoters, while H3K4me3 in combination with H3K27me3 is characteristic for poised promoters. The latter is referred to as bivalent chromatin, i.e. regions that can easily switch from a repressed status to either open or closed chromatin, well described,for example in stem and cancer cells (17,72). Thus, loss of methylation at H3K27 with a concomitant increase in H3K27 acetylation is involved in the activation of bivalent chromatin. H3K27 is acetylated by CBP/p300 (73), a cofactor shown previously to associate with MYC on chromatin (8). Therefore, we tested first whether the increase of H3K27 acetylation was paralleled by a reduction of H3K27me3, as observed for bivalent chromatin. Indeed, H3K27me3 was reduced at the promoters, whereas no change was measured in the control region (Figure 4E). The latter had very low H3K27 acetylation but high trimethylation, indicating closed chromatin (Figure 4D and E). This was also consistent with low acetylation of H3 and of H3K9, and low H3K4me3 (Figure 4A–C). The interaction of CBP/p300 with the three promoters was only partially dependent on MYC, indicating their recruitment by other factors (Figure 4F), consistent with our previous ChIP analysis (8) and with findings demonstrating CBP/p300 interaction with many different transcriptional regulators (74). Nonetheless CBP/p300 and MYC were in the same complex as determined by re-ChIP experiments, which was particularly evident for the E-box containing region of *CCND2* (Figure 3F). To expand on the observation that MYC promotes H3K27ac, we determined histone modifications that are found on promoters loaded with MYC. In these re-ChIP experiments, MYC-bound promoters showed high levels of H3K27ac as well as H3K9ac and H3K4me3, but we could not detect any H3K27me3 (Figure 4G). Thus, once MYC is bound to a promoter, the chromatin has all the hallmarks of active transcription.

In contrast to the findings with MYC and CBP/p300, the ASH2L ChIPs of the core promoters were devoid of CBP/p300, while the *CCND2* E-box region showed a robust signal (Figure 3E). This suggested that the core promoters were occupied by CBP/p300 independently of ASH2L and only partially dependent on MYC. At the *CCND2* E-box region, an interaction of the ASH2L complex with CBP/p300 was measurable. This appears to be independent of MYC as the interaction is seen under MYC low conditions and was not induced when MYC expression was turned on.

### Knockdown of ASH2L affects gene transcription and histone modification

**Figure 6.**
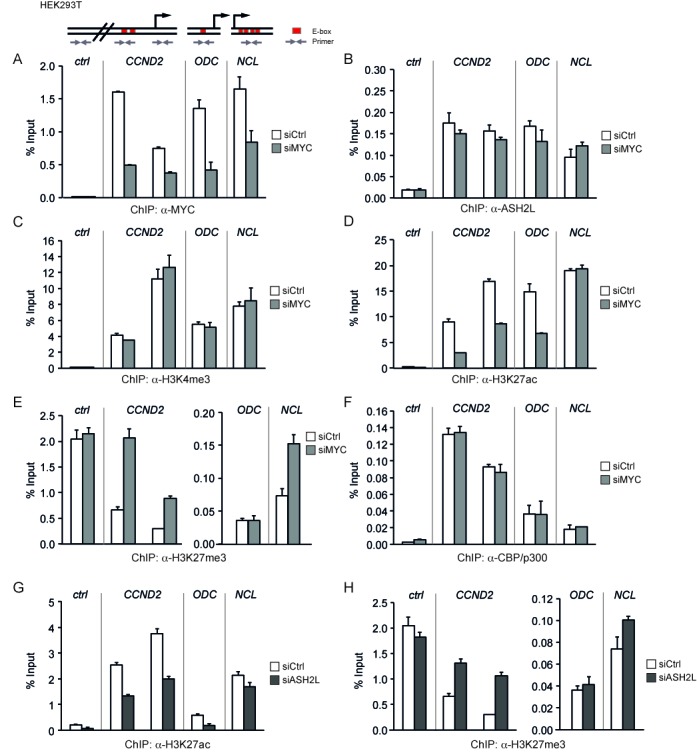
ASH2L and MYC control modification of H3K27. (**A**)–(**F**) HEK293T cells were transiently transfected with siRNA oligo pools targeting *MYC* mRNA. ChIP assays were performed with antibodies against MYC (A), ASH2L (B), H3K4me3 (C), H3K27ac (D), H3K27me3 (E) and CBP/p300 (F). DNA fragments were analyzed as described in the figures before. (**G**) and (**H**) HEK293T cells were transiently transfected with *siASH2L* oligo pools and used in ChIP assays with antibodies against H3K27ac (G) and H3K27me3 (H). The DNA fragments were amplified by qPCR with the primers indicated in the scheme. Error bars represent s.d. of PCR triplicates.

Further studies to address the interplay between MYC and ASH2L were performed in proliferating HEK293T cells using knockdown approaches. These experiments were performed with siRNA oligo pools (Figures 5 and 6). The knockdown of MYC reduced the expression of all the target genes, i.e. *CCND2*, *ODC* and *NCL*, as expected (Figure 5A and B). Similarly, the knockdown of ASH2L affected the expression of the three genes to a similar extent as the MYC knockdown. Because *MYC* expression was only slightly reduced, the expression of *CCND2*, *ODC* and *NCL* was at least partially dependent on ASH2L in these cells (Figure 5A). Reduced ASH2L levels resulted in a decrease in overall H3K4me3 and, to a lower extend, also in H3K4me2, whereas the expression of other complex members including RbBP5 and WDR5 was unaffected (Figure 5B). Upon knockdown of ASH2L, we observed a reduction of promoter bound ASH2L (Figure 5C), which was, however, less pronounced than the overall decrease of ASH2L protein levels (Figure 5B), and reduced levels of H3K4me3 (Figure 5D). The binding of RbBP5 was lowered at the *CCND2* and *ODC* promoters, but not at the *CCND2* E-box region and the *NCL* promoter (Figure 5E), while WDR5 loading was not affected (Figure 5F). Together this suggested that although ASH2L is important for H3K4 MTase activity, some of the complex components appear to be able to bind to promoters in the absence of ASH2L. This may be the consequence of other modes these proteins have to interact with chromatin (for examples see the Discussion section).

Because a correlation has been established between MYC interaction and the occurrence of H3K4me3 (75), we tested whether the presence of ASH2L was a prerequisite for MYC binding. However the knockdown of ASH2L, which resulted in a decrease in H3K4me3, did not negatively influence the loading of MYC to the three promoters (Figure 5G). Consistent with this observation was the finding that no major effect was measurable on the overall level of histone H3 acetylation (Figure 5H), which is induced upon activation of MYC in the P493-6 B cells (Figure 4B).

### ASH2L and MYC control modification of H3K27

In the P493-6 B cell model, the binding of ASH2L was independent of MYC. This was also true in HEK293T cells as the knockdown of MYC, which resulted in reduced MYC loading to the *CCND2*, *ODC* and *NCL* promoters (Figure 6A), did neither influence ASH2L binding (Figure 6B) nor H3K4me3 in any substantial manner (Figure 6C). Instead H3K27ac was reduced and H3K27me3 enhanced (Figure 6D and E), comparable to the observations in B cells. The knockdown of MYC resulted in a strong reduction of H3K27ac at the *CCND2* promoter and E-box region and at the *ODC* promoter, while no change was visible at the *NCL* promoter. Moreover, we observed an increase in H3K27me3 at the *CCND2* promoter and E-box region and at the *NCL* promoter, but not at the *ODC* promoter. Together the ratio of H3K27me3 to H3K27ac increased at all the sites analyzed when MYC was reduced. No change could be seen in the control region, which was very low in H3K27ac and high in H3K27me3 (Figure 6D and E). We did not see a reduction in CBP/p300 binding (Figure 6F), despite the effect on H3K27ac, consistent with the finding that MYC contributes little to the overall loading of these cofactors to the promoters studied both in HEK293T and in P493-6 B cells. Similar to these findings, a decrease in H3K27ac and an increase in H3K27me3 was apparent in response to the knockdown of ASH2L in HEK293T cells (Figure 6G and H). In addition to the siRNA oligo pools, we also evaluated two individual siRNA oligos targeting either *ASH2L* or *MYC*, which resulted in similar effects. The methylation of H3K27 decreased, while its acetylation increased (Supplementary Figures S6 and S7). Thus, these findings suggest that ASH2L and MYC are involved in controlling the modification of H3K27.

### MYC and WDR5, a core subunit of ASH2L complexes, co-localize at active promoters

**Figure 7.**
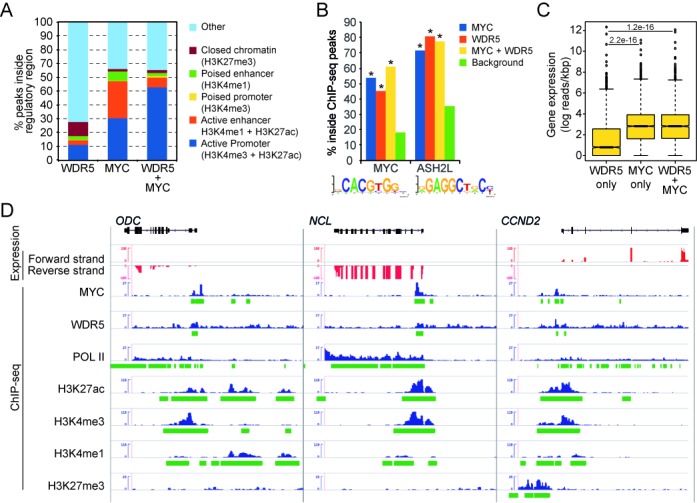
Binding of MYC and WDR5, a core subunit of ASH2L–KMT2 complexes, correlates with gene expression. (**A**) Proportion of ChIP-Seq peaks of WDR5, MYC and both MYC and WDR5 in comparison to distinct types of regulatory regions in NHEK cells. Chromatin regions were defined by the presence of ChIP-Seq peaks of distinct histone modifications or combinations thereof and the distance of the regions to transcriptional start sites. (**B**) The proportion of MYC (Jaspar Motif MA0147.1) and ASH2L (54) binding sites inside WDR5, MYC or MYC+WDR5 ChIP-Seq peaks. In all cases, the number of MYC and ASH2L-binding sites were significantly higher than binding sites in randomly selected genomic regions (*indicate conditions with a *z*-test *p*-value < 0.0001). (**C**) The log of the number of RNA reads per kilobase pairs on genomic regions close (+/- 500 bp) to WDR5, MYC and MYC+WDR5 ChIP-Seq peaks. We only considered those ChIP-Seq peaks overlapping with the 200 bp upstream regions of transcriptional start sites. (**D**) RNA-Seq-derived strand specific expression (red) and ChIP-Seq profiles (blue) and their corresponding peaks (green) of genomic regions around *ODC*, *NCL* and *CCND2*.

To further address the interaction of MYC with ASH2L complexes, we used publically available ChIP-Seq data (76). While little information is available for ASH2L and RbBP5, we used as surrogate data obtained for WDR5 (46), a subunit of the core complexes containing KMT2 MTases (19), and a protein that binds to MYC (Supplementary Figure S3). WDR5 may interact with chromatin independently of ASH2L, a note that has to be kept in mind when using WDR5 as a surrogate of ASH2L. In NHEKs, ChIP-Seq peaks of MYC are predominantly found at active enhancers and promoters (26% and 30%, respectively), which are defined by genomic regions carrying H3K4me1/H3K27ac and H3K4me3/H3K27ac, respectively (Figure 7A). In contrast, WDR5 ChIP-Seq peaks localize to a broad variety of different sites that are categorized as active enhancers and promoters only to a small extend (3% and 11%, respectively). However, when the peaks with co-localization of MYC and WDR5 are analyzed, 53% of the sites are positive for H3K4me3/H3K27ac and thus classify as active promoters. In this data set, 86% of the active promoters defined as described above score positive for POL II binding (Supplementary Table S2), while only few poised promoters (6%), poised enhancers (3%), active enhancers (16%) and repressive domains (0.1%) regions reveal POL II binding. Together these findings support our functional data indicating that MYC and ASH2L complexes co-localize at transcribed promoters.

Although the available data did not allow the analysis of ASH2L directly, we made use of the recently reported DNA consensus sequence that is recognized by ASH2L (54). This allowed us to evaluate further the interaction of MYC and ASH2L. The ChIP-Seq peaks of either MYC or WDR5 were expanded in both directions by 125 base pairs and the corresponding sequence interval was searched for MYC- and ASH2L-binding sites (Figure 7B). More than 50% of the MYC-binding regions contained a sequence similar to a CACGTG-type E-box (Figure 7B). 45% of WDR5 sites also contained such E-boxes and 61% of the regions that contained both MYC and WDR5 had E-boxes. These findings provide additional evidence that MYC and ASH2L complexes coexist on chromatin. This is further supported by the observation that ASH2L-binding motifs co-localize with MYC- and WDR5-binding sites (Figure 7B).

Finally, we addressed the question whether the ChIP-Seq peaks of MYC and WDR5, as a surrogate of ASH2L complexes, have an influence on gene expression whenever they are located close to gene promoters, i.e. within 200 base pairs 5’ of the start of transcription (TSS). Using public RNA-Seq data from NHEK cells, the analysis revealed that genes with MYC or MYC/WDR5 ChIP-Seq peaks were significantly higher expressed than genes with WDR5 ChIP-Seq peaks alone (Figure 7C). This argued for many WDR5 ChIP-Seq peaks that are not close to promoters, which is supported by the low overlap between WDR5 and promoters, either active or poised (Figure 7A and C). Together this analysis on the global distribution of MYC and WDR5, which is a core component of ASH2L–KMT2 complexes, support our biochemical and functional data indicating that MYC and ASH2L complexes cooperate in controlling gene expression.

The data on NHEK cells used above were also analyzed for the three genes, i.e. *ODC*, *NCL* and *CCND2*, studied in HEK293T and in P493-6 B cells. All three genes were expressed and showed POL II binding (Figure 7D). The core promoter regions and the 5’ coding sequences of all three genes revealed high H3K4me3 and H3K27ac and very low H3K4me1 and H3K27me3. This correlated with the major and minor MYC ChIP-Seq peaks in *NCL* and *ODC*, respectively. The major MYC ChIP-Seq peaks in *ODC* and *CCND2* were more 5’ relative to the core promoter and displayed high H3K27ac and lower H3K4me3 compared to the core promoter. Again these regions showed very low H3K4me1 and H3K27me3. The binding of WDR5 was very broad and distributed over the entire loci of all three genes. Nevertheless increased WDR5 ChIP-Seq signals were associated with the MYC ChIP-Seq peaks. Together these observations support well the findings obtained for HEK293T and P493-6 B cells, defining MYC together with KMT2 complexes as closely linked with active chromatin.

## DISCUSSION

We have identified the trithorax protein ASH2L as a direct interaction partner of the oncoprotein MYC. Our findings suggest that MYC recruits through ASH2L and possibly WDR5 MTase complexes that are capable of trimethylating H3K4 (Figures 1 and 2). Our binding experiments suggest that ASH2L interacts directly with MYC through a region N-terminal of the bHLHZip domain. We also identified the HLHZip as an indirect interaction domain. Thus, the ASH2L–KMT2 complex appears to have additional regions that can bind to MYC. Indeed, we observed that WDR5 also directly interacts with MYC and that the CTD of MLL2 co-IPs efficiently with MYC (Supplementary Figure S3). Thus, it appears that MYC has multiple contact sites with the KMT2 complex and provides an explanation for the indirect association of ASH2L with the HLHZip domain of MYC.

An interaction of MYC with ASH2L was also measurable on chromatin (Figure 3). Unlike our expectation, MYC did not recruit ASH2L to the three studied MYC-responsive promoters, i.e. *CCND2*, *ODC* and *NCL*. Instead ASH2L and other core components of KMT2 complexes were bound to these promoters prior to the presence of MYC (Figures 3 and 6). In support, genome-wide studies have indicated that MYC binds preferentially to regions in the genome that bear histone marks associated with transcribed promoters, including H3K4me3 (75). The knockdown of ASH2L, which resulted in reduced ASH2L and RbBP5 binding and a reduction of H3K4me3, did not affect MYC binding to promoters (Figure 5). It should be noted that despite a substantial reduction in ASH2L protein levels, the ASH2L-specific ChIP reported only a modest reduction of promoter-associated ASH2L (Figure 5). Thus, it appears that the reduction of ASH2L at promoters is less prominent than the overall reduction of the protein, indicating efficient recruitment of ASH2L to chromatin. Together these findings are compatible with a previous interpretation that MYC/MAX complexes require open chromatin for binding to specific DNA sites (75). The recruitment of MYC was also not involved in modulating the level of H3K4me3 but induced the expression of the three genes analyzed (Figures 4 and 6, and Supplementary Figure S4). Their expression correlated with an exchange of H3K27me3 with H3K27ac at these promoters (Figures 4 and 6). This suggests that MYC together with ASH2L controls the modification of H3K27.

Trimethylation of H3K27 is catalyzed by the Polycomb-repressive complex 2 (PRC2), which together with other Polycomb group proteins negatively controls gene transcription (20). EZH2, a core subunit of PRC2, is the SET domain protein that trimethylates H3K27, a mark that is associated with transcriptional repression. Together with H3K4me3, H3K27me3 defines so-called bivalent chromatin, which poises the expression of many genes (17,77). Bivalent chromatin was originally identified in stem cells (78,79), but has been observed more recently in many other cell types, including tumor cells (e.g. (80–83)). Methylation at K27 is antagonized by acetylation, requiring first demethylation that is carried out by at least two enzymes, UTX (also KDM6A) and JMJD3 (also KDM6B) (84,85). UTX has been found in some ASH2L-containing KMT2 complexes (85,86), indicating that these are involved in controlling bivalent chromatin. Active chromatin carries H3K27ac, which can be catalyzed by the CBP/p300 acetyltransferases (73,87). Together these findings imply that the interplay between PRC2, KMT2 complexes and CBP/p300 control bivalent chromatin. Our findings lead to the postulate that MYC has two molecular functions in coordinating the modification of H3K27. First, we postulate that MYC activates a KMT2 complex-associated demethylase, which results in the demethylation of H3K27me3 and thus in the generation of a substrate for acetyltransferases. Second, MYC stimulates CBP/p300, which are known to acetylate H3K27. In summary, these two functions are capable to induce a switch from bivalent to active chromatin and may explain a role of MYC as broad activator of gene transcription (88,89). A recent study has demonstrated that p53 also cooperates with an H3K4me3 MTase and p300, providing further evidence for the importance of the combination of H3K4me3 and H3K27ac for gene transcription (90).

As pointed out above, bivalent chromatin regulates gene transcription also in tumor cells (17). In support of this notion, several factors that control bivalent chromatin have been found associated with cancer. For example, overexpression of EZH2 is associated with many different tumors, including breast and prostate cancer (91,92). Also several MLLs are involved in cancer, as in MLLs the *MLL1* gene is the translocation partner of more than 100 genes. Moreover in other tumors, some *MLLs* are amplified (93,94). Furthermore, we have observed that ASH2L cooperates with Ha-RAS in transforming rodent cells and that the protein is overexpressed in many human tumors of different entity (38,60). Finally, also CBP and p300 have been linked to tumorigenesis (95,96). Thus, both the modifications that occur at H3K4 and H3K27 and the enzymes catalyzing these modifications are linked to cancer. It will now be important to understand how MYC, which regulates these processes, cooperates with the above-mentioned tumor-associated alterations.

The mechanism of how bivalent chromatin is controlled rests on the interplay of different enzymes recruited to specific sites in DNA. The acetyltransferases CBP and p300 are recruited by numerous transcriptional regulators (74), one being MYC (8), which is consistent with the finding that these enzymes mark mainly enhancer sequences, in addition to promoters (31). PRC2 and ASH2L–KMT2 complexes appear to have multiple ways to interact with chromatin (17,77). Because we have identified ASH2L as a direct interaction partner of MYC in this study, it is worth considering how ASH2L–KMT2 complexes are thought to interact with chromatin. Two distinct mechanisms have been suggested.

The first involves the recruitment of ASH2L complexes by transcriptional regulators to chromatin in a sequence-specific manner. The second indicates that ASH2L–KMT2 complexes interact with chromatin components, for example specifically modified core histones, which allows context-dependent binding. These two possibilities, which are detailed below, are fundamentally different as in the first the information to position the complex is present in the DNA sequence, while in the second the binding occurs to specialized chromatin. Thus the second possibility allows in principle DNA sequence-independent localization at and modification of chromatin. As to the first possibility, several transcription factors interact with ASH2L–KMT2 complexes and thus can potentially recruit H3K4 trimethylase activity to specific sites in chromatin. These include the broadly acting NF-Y (97), NF-E2 that regulates the β-globin locus (98,99), Mef2d that controls muscle-specific genes (100–102) and TBX1 that plays an important role in heart development (103). APδ recruits an ASH2L complex to regulate *Hoxc8* and *Fgfr3* (104,105), and Pax7 regulates *Myf5* expression upon Carm1-dependent methylation, which in turn allows recruitment of an ASH2L complex (106,107). In these examples, the recruitment of ASH2L–KMT2 complexes is associated with increased H3K4me3, suggesting that these transcriptional regulators target ASH2L-associated MTases to specific genes. Thus, these transcription factors seem to function differently from MYC. It will be interesting to test whether they also affect the modification of H3K27.

In addition ASH2L–KMT2 complexes bind to chromatin independent of defined DNA sequences, as outlined as second possibility. It appears that several subunits of ASH2L–KMT2 complexes display distinct activities compatible with binding to chromatin. WDR5 interacts with the N-terminus of histone H3 (108–112), a function that is negatively regulated by methylation of H3R2 (63), and thus positions the complex close to the K4 acceptor site. MLLs possess also affinity for chromatin, for example the PHD domain of MLL1 interacts with core histones, with a preference for di- and trimethylated H3K4 (113), postulating that the complex may perpetuate the H3K4me3 mark. Moreover, recent findings suggest that ASH2L itself has DNA-binding capacity (54,114), although the sequence specificity is only weak and may not be sufficient to position the complex to specific sites on the DNA independent of any other interactions. Additional subunits, some only associated with selected ASH2L–KMT2 complexes, may also contribute to target KMT2 group MTases to specific regions in the chromatin. Thus, multiple possibilities exist that allow the ASH2L–KMT2 complexes to bind to chromatin without requiring sequence-specific transcription factors. Nevertheless, the activities associated with these complexes, this includes the H3K4 MTase and the H3K27 demethylase functions, need to be regulated. We would expect that these activities are controlled, at least in part, by sequence-specific transcription factors, as demonstrated here for MYC. We also notice that there is a difference between the recruiting factors, such as NF-E2, and the regulating factors, such as MYC. The former seem to affect primarily the methylation of H3K4, while MYC modulates H3K27 modification but not H3K4. The interplay and the regulation of gene transcription are also implicated by analyzing the functional consequences of MYC with WDR5, a core component of the ASH2L complex and a binding partner of MYC. Together MYC and WDR5 define active promoters that are characterized by POL II loading and transcription of the associated genes (Figure 7).

**Figure 8.**
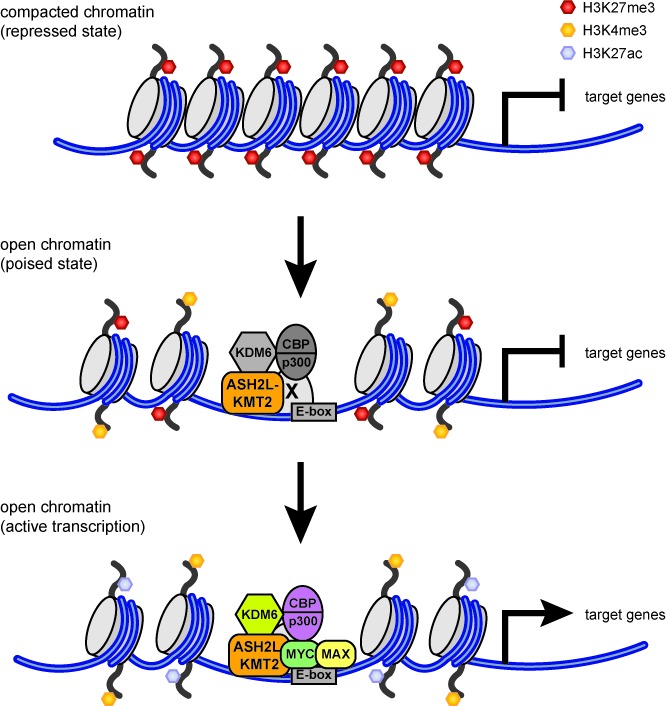
Schematic and simplified depiction of the interaction of MYC with ASH2L–KMT2 complexes and their effects on core histone modifications and gene transcription. In the repressed state, characterized by H3K27me3, no ASH2L–KMT2 complexes are bound to chromatin. In open chromatin, specific promoters are associated with ASH2L–KMT2 complexes resulting in high H3K4me3. Two forms of open chromatin are indicated, one is characterized by H3K27me3 in addition to H3K4me3. This is referred to as bivalent chromatin and promoters with these two marks are typically poised. How ASH2L–KMT2 complexes are recruited initially is not well understood. This may occur through direct interaction of the KMT2 complex with chromatin or through an unknown transcription factor (referred to as X). KDM6 enzymes, which demethylate H3K27, and CBP/p300, which acetylate H3K27, have been reported to interact with KMT2 complexes and may therefore be located on promoters prior to MYC binding. In the absence of MYC, H3K27 modifying enzymes seem to be largely inactive. Promoters switch to a transcriptionally active mode upon binding of MYC, which we postulate to result in the activation of these H3K27 modifying enzymes (indicated by the color change). This leads to a reduction of H3K27me3 and concomitant increase in H3K27ac, which combined with H3K4me3 marks open chromatin with active gene transcription.

In summary our studies provide evidence that MYC, together with ASH2L–KMT2 complexes, controls gene transcription by activating bivalent chromatin (Figure 8). This suggests a novel, additional mechanism how MYC controls gene expression. It will now be interesting to define how MYC affects the enzymes associated with methylation and demethylation of H3K27.

## SUPPLEMENTARY DATA

Supplementary Data are available at NAR Online.

SUPPLEMENTARY DATA
