# Supplementary material for: The interaction of MYC with the trithorax protein ASH2L promotes gene transcription by regulating H3K27 modification
Source: Nucleic Acids Res. 2014 Apr 29;42(11):6901–20. doi: 10.1093/nar/gku312 (PMC4066752; doi:10.1093/nar/gku312)
Supplement: SUPPLEMENTARY DATA [file supp_42_11_6901__index.html]

The interaction of MYC with the trithorax protein ASH2L promotes gene transcription by regulating H3K27 modification — The interaction of MYC with the trithorax protein ASH2L promotes gene transcription by regulating H3K27 modification — SUPPLEMENTARY DATA 

# The interaction of MYC with the trithorax protein ASH2L promotes gene transcription by regulating H3K27 modification

## SUPPLEMENTARY DATA

**Files in this Data Supplement:**

- SUPPLEMENTARY DATA
