# Supplementary material for: The interaction of MYC with the trithorax protein ASH2L promotes gene transcription by regulating H3K27 modification
Source: Nucleic Acids Res. 2014 Apr 29;42(11):6901–20. doi: 10.1093/nar/gku312 (PMC4066752; doi:10.1093/nar/gku312)

SUPPLEMENTARY INFORMATION

The interaction of MYC with the trithorax protein ASH2L promotes gene transcription by regulating H3K27 modification

Andrea Ullius[1], Juliane Lüscher-Firzlaff[1], Ivan G. Costa[2], Gesa Walsemann[1,4], Alexandra H. Forst[1,4], Eduardo G. Gusmao[2], Karsten Kapelle[1,4], Henning Kleine[1,4], Elisabeth Kremmer[3], Jörg Vervoorts[1,5], Bernhard Lüscher[1,5,6]

[1]Institute of Biochemistry and Molecular Biology, [2]IZKF Research Group Computational Biology, Medical School, RWTH Aachen University, 52074 Aachen, Germany; [3]Helmholtz Zentrum München, Institut für Molekulare Immunologie, Marchioninistr. 25, 81377 München, Germany

[4]Present addresses: PAREXEL International GmbH, Berlin, Germany (GW); Abbvie GmbH & Ko.KG, Wiesbaden, Germany (AHF and HK); Elchrom Scientific, Cham, Switzerland (KK).

[5]These author contributed equally

SUPPLEMENTARY FIGURE LEGENDS

Supplementary Figure S1

ASH2L-specific antibodies.

(A) HA-tagged ASH2L was expressed in COS7 cells. ASH2L proteins were immunoprecipitated using the polyclonal rabbit serum 548 (i) or HA-specific antibodies. Pre-immune serum (pi) and the MAD1-specific mAb 5F4 were used as controls. Total lysates of cells transfected with the HA-ASH2L expression plasmid (input) or with empty vector (input control) were loaded. The Western blot (WB) was probed with an antibody specific for the HA-tag (3F10).

(B) ASH2L was immunoprecipitated from lysates of Jurkat T cells using serum 548 (as in panel A), and mAbs 4B5 and 4C5. For control the lysates were incubated with Protein-G agarose. Input shows total cell lysate. The Western blot was developed using the ASH2L-specific polyclonal rabbit serum 549.

(C) The indicated proteins were immunoprecipitated from lysates of Jurkat T cells. The co-immunoprecipitation of ASH2L was analyzed by immunoblotting using the ASH2L-specific 548-8 polyclonal antiserum. Antibodies specific for the HA-tag were used as negative control.

(D) MYC was immunoprecipitated from HEK293 cells using IgG as control antibodies. The co-immunoprecipitation of ASH2L was analyzed by immunoblotting using the ASH2L-specific 4C5 monoclonal antibody.

Supplementary Figure S2

Mapping of the MYC and ASH2L interaction domains.

(A,B) Glutathione-S-transferase (GST)-ASH2L and -MAX fusion proteins were used to detect interactions with in vitro transcribed and translated MYC wt and deletion mutants as indicated, GST alone was used as control. One-tenth of the in vitro transcribed/translated material was loaded as control (Input (1/10)).

(C) HEK293 cells were transiently transfected with plasmids expressing Flag-tagged MYC wt and deletion mutants or empty vector control. After immunoprecipitation of MYC proteins from F-buffer lysates using Flag-specific antibodies endogenous, co-immunoprecipitated ASH2L was detected by immunoblotting with the ASH2L-specific mAb 4C5 (upper panel). The expressions of the different Flag-tagged MYC mutants were detected by immunoblotting of whole cell lysates using the Flag-specific mAb M2 (lower panel).

(D) HEK293 cells were co-transfected with Flag-tagged ASH2L and different HA-tagged MYC deletion mutants or empty vector as control. After immunoprecipitation of ASH2L from F-buffer lysates using HA-specific antibodies co-immunoprecipitated MYC was detected by immunoblotting with the Flag-specific mAb M2 (upper panel). The expressions of the different HA-tagged MYC mutants and Flag-tagged ASH2L is shown by immunoblotting of whole cell lysates using the HA-specific mAb 3F10 (middle panel) and the Flag-specific mAb M2 (bottom panel), respectively.

Supplementary Figure S3

(A) ASH2L, RbBP5, and WDR5 were transcribed and translated in vitro and the fraction bound to GST-MYC-263-439 and GST alone analyzed. One-tenth of the in vitro

transcribed/translated material was loaded as input control. The respective input of the GST proteins is shown by the Coomassie Blue (CB) stained gel.

(B) Binding of recombinant His_6-WDR5 to GST-MYC-263-439 and GST alone were analyzed by GST-pull-down experiments and immunoblotting of WDR5, respectively. One-tenth of His_6-WDR5 was loaded as input control. The respective input of GST proteins is shown by Ponceau staining.

(C) HEK293T cells were transfected with MYC and Flag-tagged MLL2 fragment (653 aa of C-terminus), WDR5, RbBP5, or ASH2L. Cells were harvested 48 hours after transfection and lysed in co-IP buffer. MYC was immunoprecipitated with MYC-specific antibodies (N262) and precipitates were analyzed by Western blotting with α-Flag antibodies. Whole cell lysates were subjected to Western blot analysis using Flag- and MYC-specific (N262) antibodies. The arrow head in the upper panel indicates RbBP5.

Supplementary Figure S4

Characterization of P493-6 B cells.

P493-6 cells were treated with 0.1 μg/ml tetracycline for 72 hours. Removal of tetracycline was achieved by washing the cells with PBS and adding fresh RPMI medium containing 10% FCS. Cells were harvested after the addition of fresh medium without tetracycline at the indicated time points. For control cells were grown in the absence of tetracycline and analyzed in parallel (Ø).

(A) Expression of *MYC* mRNA was analyzed by quantitative RT-PCR and normalized to the expression of *GUS*. The relative expression of the normalized *MYC* mRNA is shown with the value set to 1 at the 0 hour time point. Whole cell lysates of P493-6 cells were prepared 0, 3, and 6 hours after tetracycline removal and MYC protein levels were measured by Western Blot analysis using MYC-specific antibodies (N262) and actin as loading control (inset).

(B) P493-6 were fixed with methanol and stained with propidium iodide. Distribution of cells in G1, S or G2/M phase was determined using flow cytometry analysis (FACS).

(C) Expression of *CCND2*, *ODC*, and *NCL* mRNA was analyzed at the different time points by quantitative RT-PCR and normalized to *GUS*. The relative expression of the normalized *CCND2*, *ODC*, and *NCL* mRNAs is shown with the values set to 1 at the 0 hour time point.

Supplementary Figure S5

(A) P493-6 B cells were treated with 0.1 μg/ml tetracycline for 72 hours. ChIP assays were performed either directly (-MYC) or six hours after tetracycline removal (+MYC) with IgG control antibodies. Immunoprecipitated DNA was amplified by quantitative PCR (qPCR) with primers for *CCND2*, *ODC*, and *NCL* promoter regions and a control region 22 kbp upstream of the *CCND2* promoter (*ctrl*), as indicated in the scheme.

(B) HEK293T cells were transiently transfected with siRNA oligo pools targeting *ASH2L* mRNA or with a control oligo pool (Ctrl). ChIP assays were carried out with IgG control antibodies. Purified DNA was analyzed by qPCR as described in A.

(C) HEK293T cells were transiently transfected with siRNA oligo pools targeting *MYC* mRNA or with a control oligo pool (Ctrl). ChIP assays were performed with IgG control antibodies. DNA fragments were analyzed as described in A.

Supplementary Figure S6

ChIP assays of HEK293T cells, transfected with single siRNA oligos (#1 in panels A, C, E, and G or #2 in panels B, D, F, and H) targeting *ASH2L* mRNA or with a control siRNA oligos (Ctrl), were performed with antibodies against ASH2L (A+B), H3K27ac (C+D), H3K27me3 (E+F), and IgG as control (G+H). Immunoprecipitated DNA was amplified by quantitative PCR (qPCR) with primers for *CCND2*, *ODC*, and *NCL* promoter regions and a control region 22 kbp upstream of the *CCND2* promoter (*ctrl*), as indicated in the scheme.

Supplementary Figure S7

ChIP assays of HEK293T cells, transfected with single siRNA oligos (#1 in panels A, C, E, and G or #2 in panels B, D, F, and H) targeting *MYC* mRNA or with a control siRNA oligos (Ctrl), were performed with antibodies against MYC (A+B), H3K27ac (C+D), H3K27me3 (E+F), and IgG as control (G+H). Immunoprecipitated DNA was amplified by quantitative PCR (qPCR) with primers for *CCND2*, *ODC*, and *NCL* promoter regions and a control region 22 kbp upstream of the *CCND2* promoter (*ctrl*), as indicated in the scheme.

SUPPLEMENTARY TABLES

Supplementary Table S1 – Statistics on TF Chip-Seq Peaks

	Poised Enhancer	Closed Chromatin	Active Gene	Active Enhancer	Poised Gene	Total Peaks
WDR5	1670	6170	6706	2056	298	61360
MYC	2484	616	11298	10039	173	37342
MYC and WDR5	235	192	5209	730	89	9894

Supplementary Table S2 – Statistics of Regulatory Regions and POL II

	no POL II	POL II	Total
Poised Promoter	664 (94%)	39 (6%)	703
Poised Enhancer	47164 (97%)	1461 (3%)	48625
Active Promoter	1706 (14%)	8754 (86%)	10460
Active Enhancer	26365 (84%)	9865 (16%)	36230
Repressive Marks	53214 (99.9%)	114 (0.1%)	53328

Supplementary Table S3 – Binding Site Statistics

Peaks	Motif	Prop. Sites	Prop. Sites Back.	P-value	Sites on Peaks	Sites on Back
MYC	MYC	53.6	17.83	10^{-16}	16968	6315
MYC	ASH2L	71.53	35.4	10^{-16}	18717	10837
MYC and WDR5	MYC	61	18.1	10^{-16}	6693	2037
MYC and WDR5	ASH2L	77.28	35.95	10^{-16}	6757	3449
WDR5	MYC	45.34	18.61	10^{-16}	23624	11042
WDR5	ASH2L	80.56	35.24	10^{-16}	35822	18135

Figure S1 revised

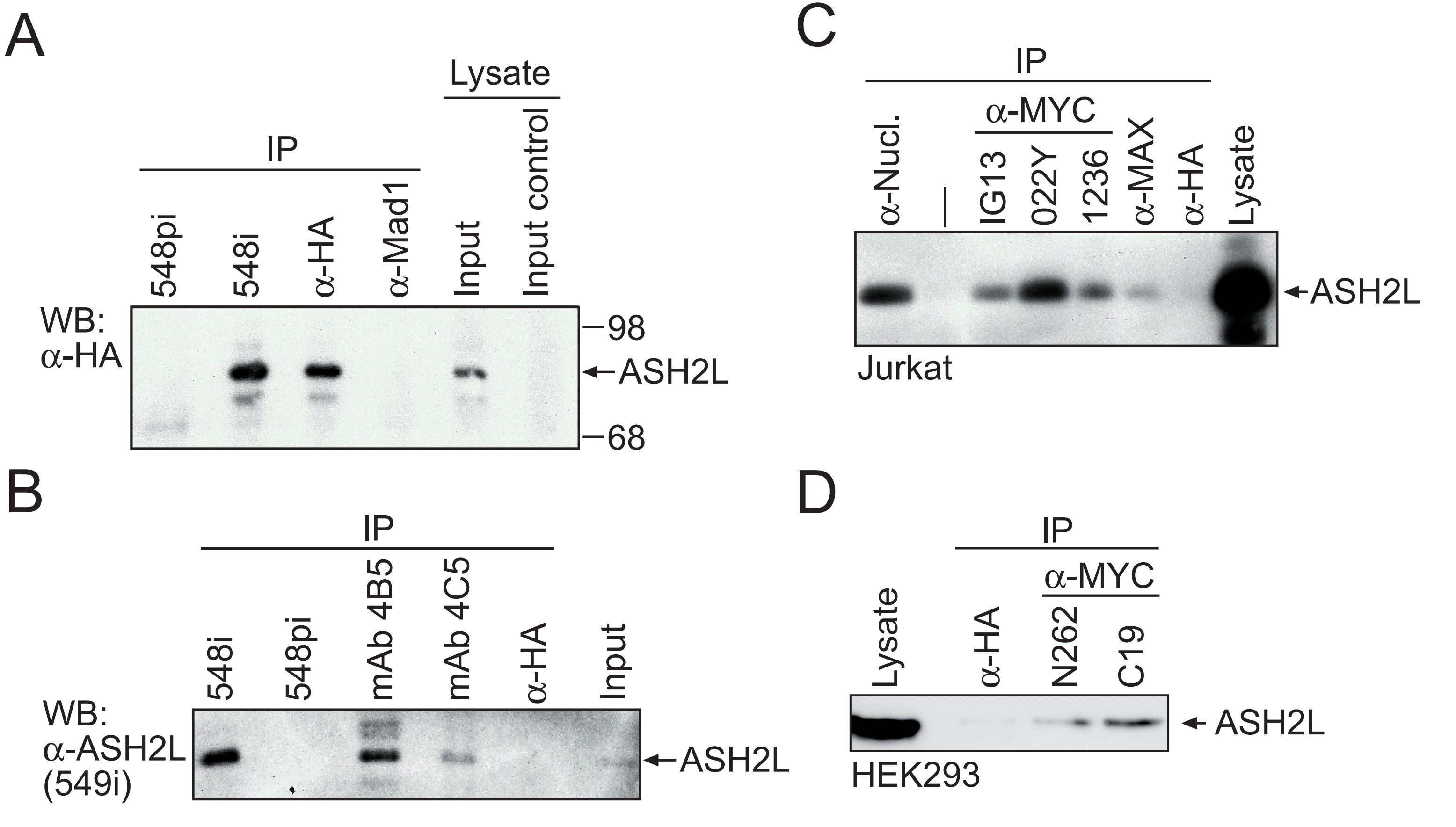

Figure S2 revised

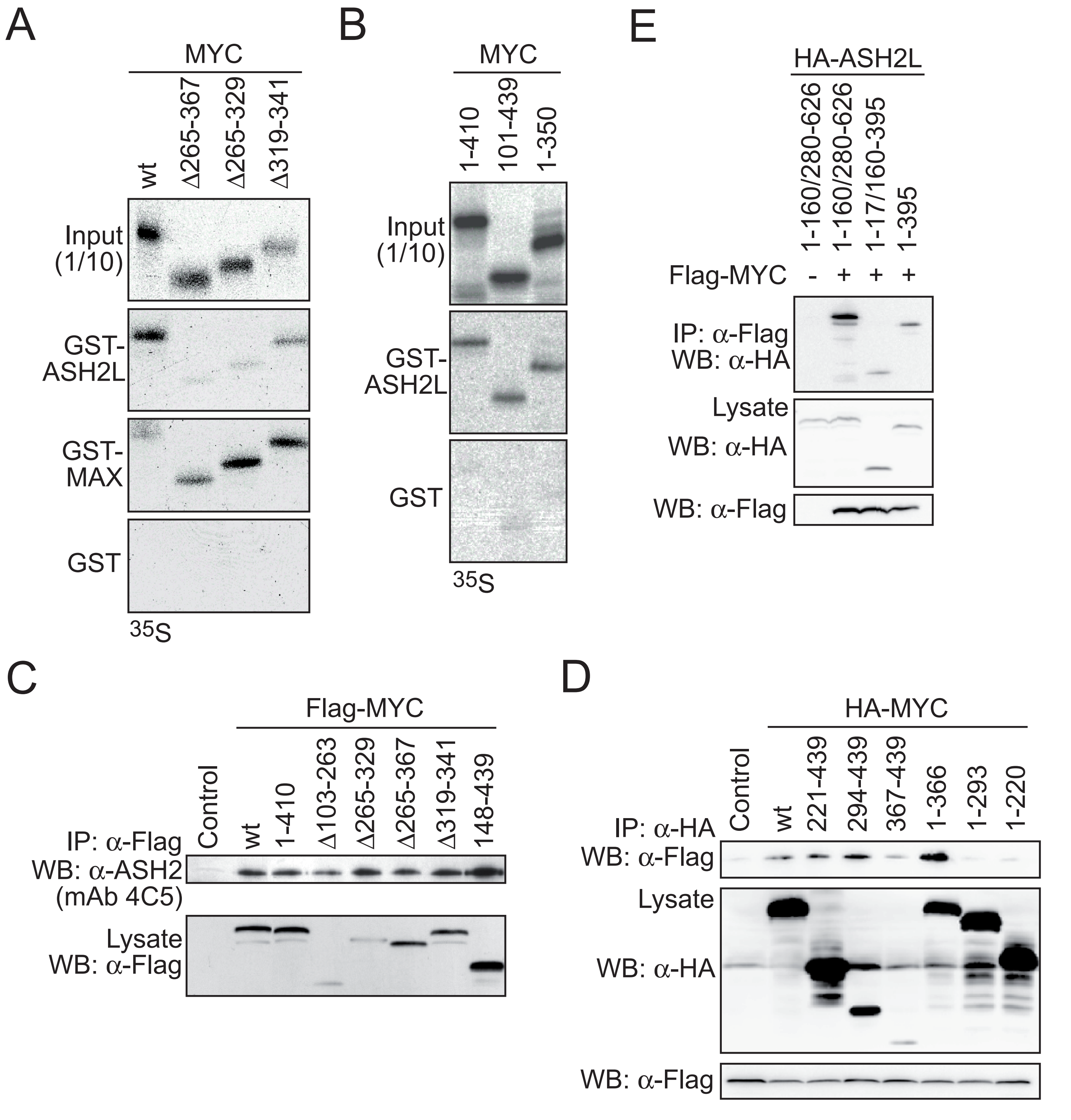

Figure S3 revised

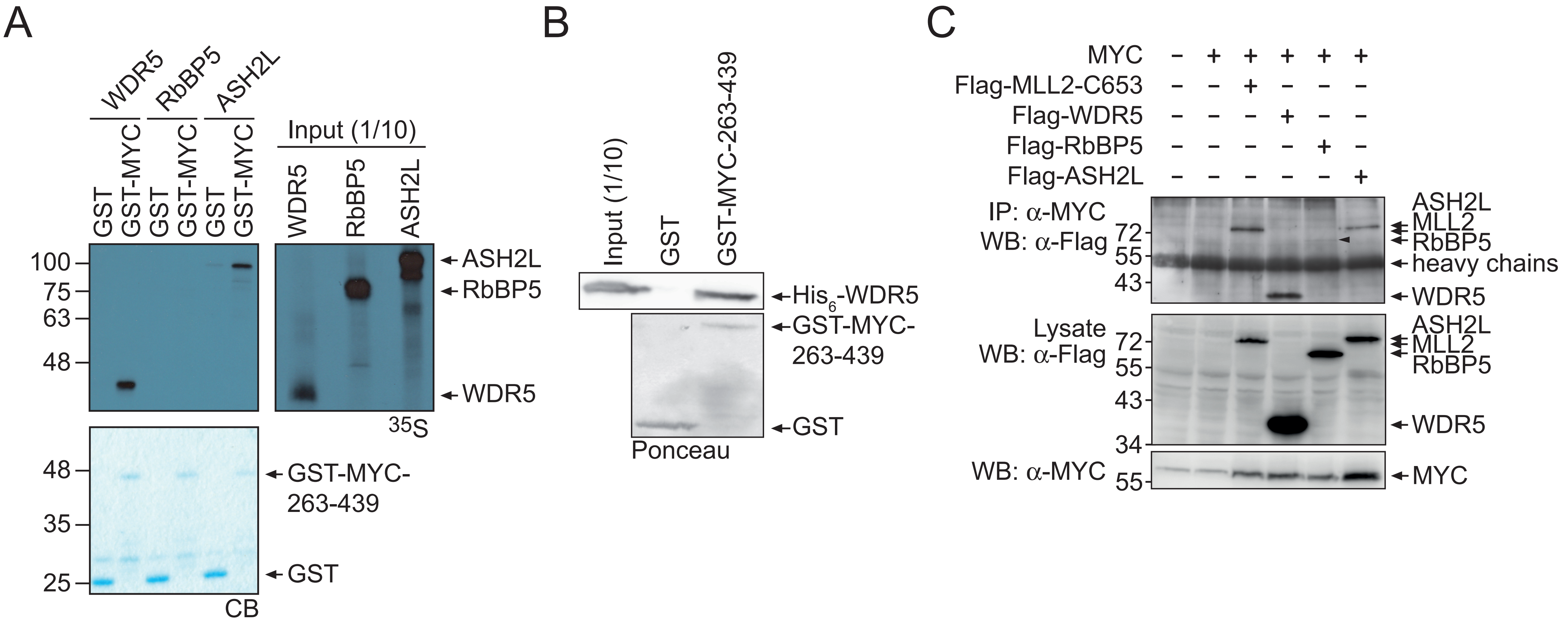

Figure S4 revised

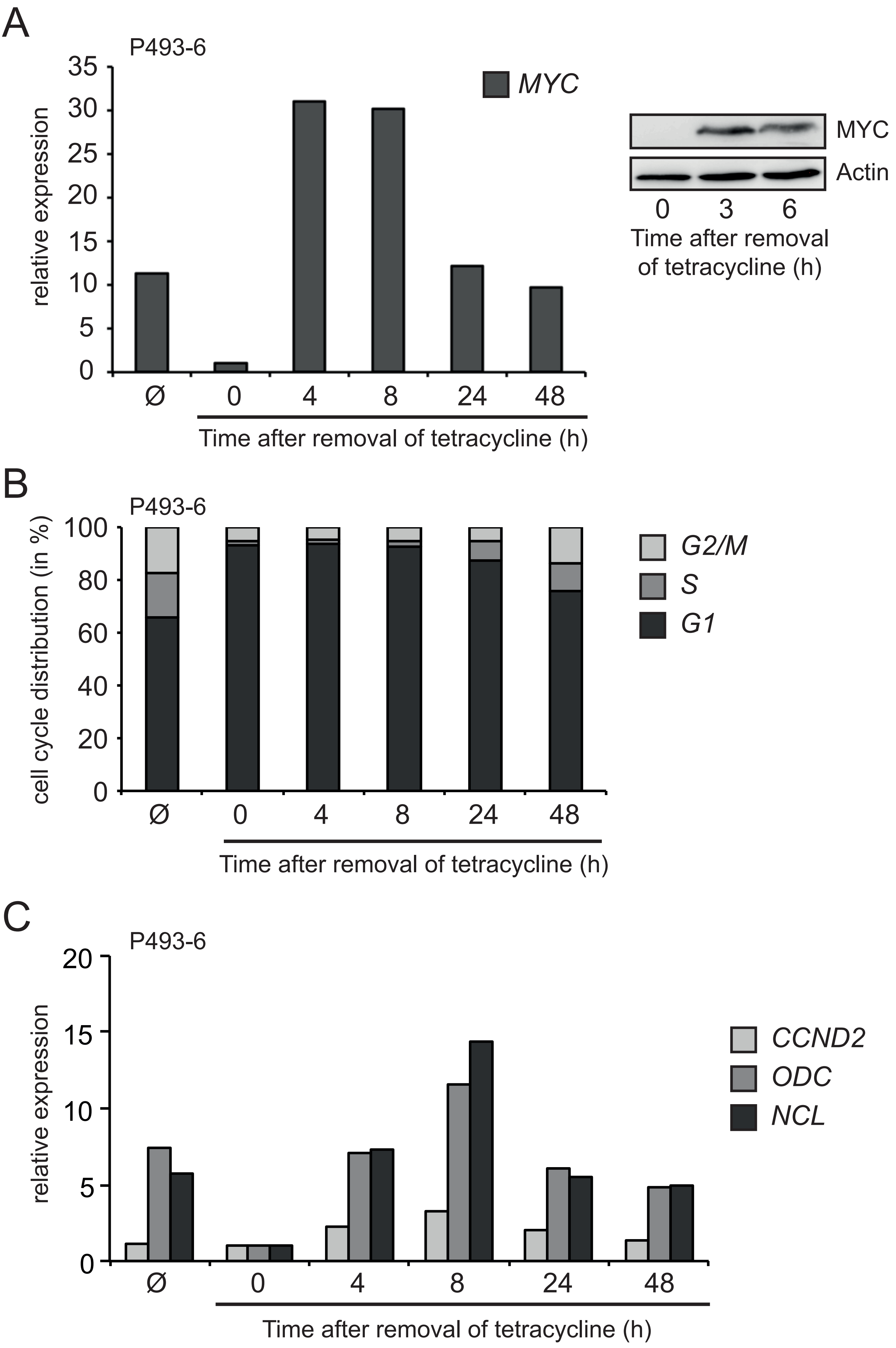

Figure S5 revised

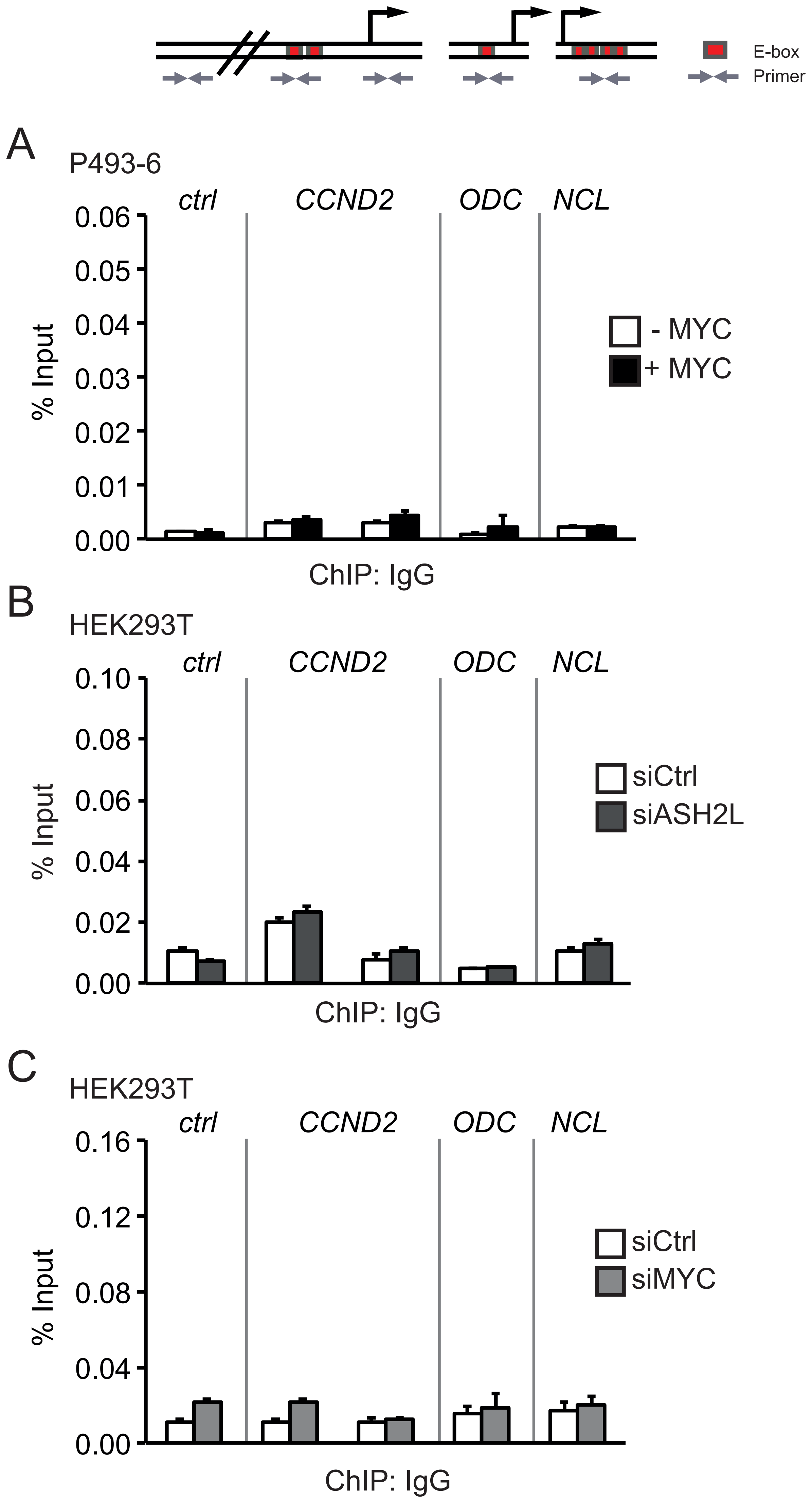

Figure S6 revised

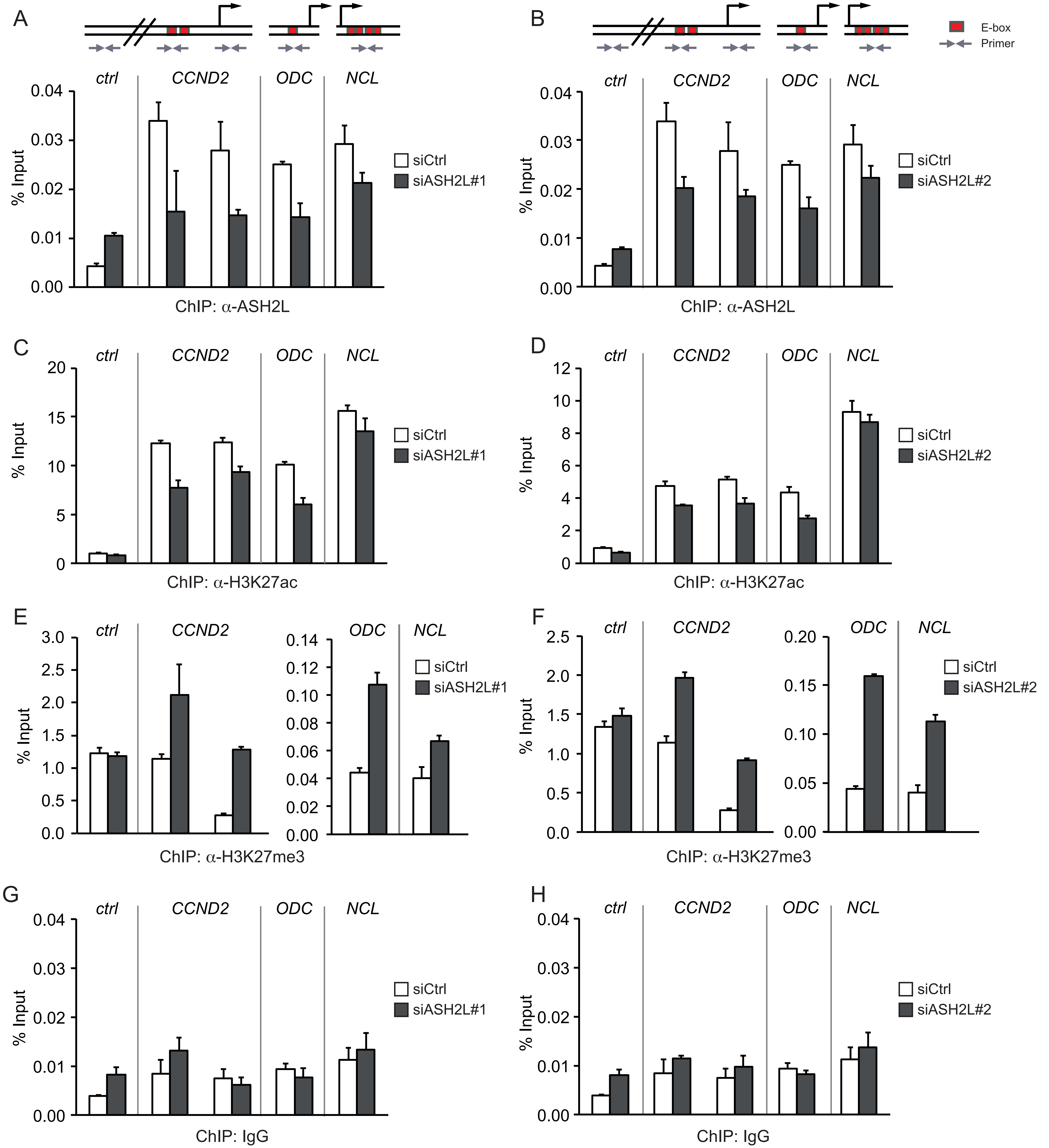

Figure S7 revised

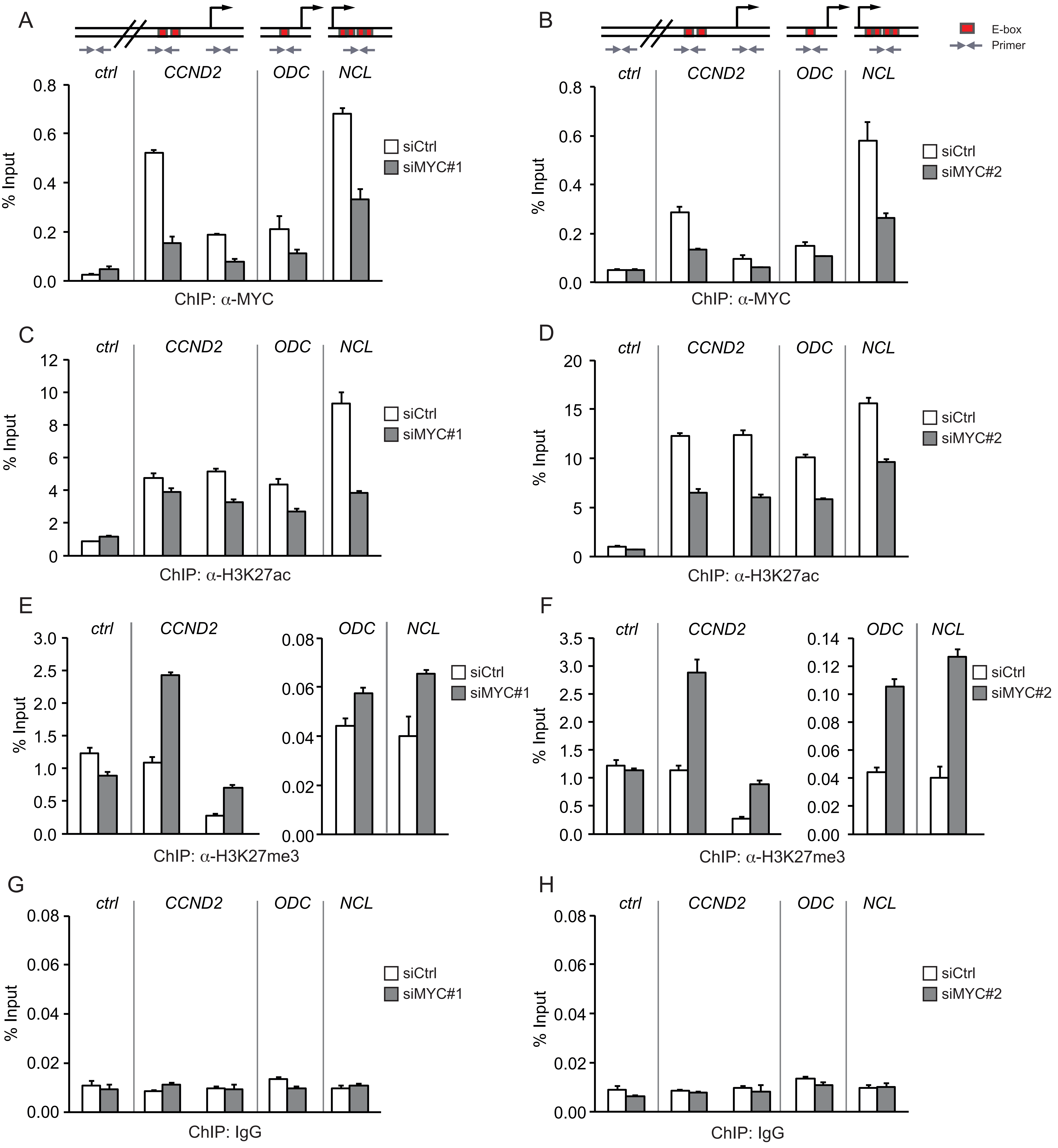

Supplement: SUPPLEMENTARY DATA [file supp_gku312_nar-02550-x-2013-File010.pdf]
